# Three-dimensional images reveal the impact of the endosymbiont *Midichloria mitochondrii* on the host mitochondria

**DOI:** 10.1038/s41467-023-39758-x

**Published:** 2023-07-12

**Authors:** Zerrin Uzum, Dmitry Ershov, Michael J. Pavia, Adeline Mallet, Olivier Gorgette, Olivier Plantard, Davide Sassera, Fabrizia Stavru

**Affiliations:** 1 Unit Evolutionary Biology of the Microbial Cell, Institut Pasteur; CNRS UMR2001, Paris, France; 2 Image Analysis Hub, Cell Biology and Infection Department, Institut Pasteur, Paris, France; 3 Bioinformatics and Biostatistics HUB, Department of Computational Biology, Institut Pasteur, USR 3756 CNRS, Paris, France; 4 School of Life Sciences, Arizona State University, Tempe, AZ USA; 5 Ultrastructural BioImaging Core Facility, Institut Pasteur, Paris, France; 6 INRAE, BIOEPAR, Oniris, F-44300 Nantes, France; 7 Department of Biology and Biotechnology, University of Pavia, Pavia, Italy

**Keywords:** Symbiosis, 3-D reconstruction, Mitochondria

## Abstract

The hard tick, *Ixodes ricinus*, a main Lyme disease vector, harbors an intracellular bacterial endosymbiont. *Midichloria mitochondrii* is maternally inherited and resides in the mitochondria of *I. ricinus* oocytes, but the consequences of this endosymbiosis are not well understood. Here, we provide 3D images of wild-type and aposymbiotic *I. ricinus* oocytes generated with focused ion beam-scanning electron microscopy. Quantitative image analyses of endosymbionts and oocyte mitochondria at different maturation stages show that the populations of both mitochondrion-associated bacteria and bacterium-hosting mitochondria increase upon vitellogenisation, and that mitochondria can host multiple bacteria in later stages. Three-dimensional reconstructions show symbiosis-dependent morphologies of mitochondria and demonstrate complete *M. mitochondrii* inclusion inside a mitochondrion. Cytoplasmic endosymbiont located close to mitochondria are not oriented towards the mitochondria, suggesting that bacterial recolonization is unlikely. We further demonstrate individual globular-shaped mitochondria in the wild type oocytes, while aposymbiotic oocytes only contain a mitochondrial network. In summary, our study suggests that *M. mitochondrii* modulates mitochondrial fragmentation in oogenesis possibly affecting organelle function and ensuring its presence over generations.

## Abstract (web summary)

The mitochondrial symbiont, *Candidatus* Midichloria mitochondrii, exists in the hard tick *Ixodus ricinus*, the main vector for Lyme disease. Here, the authors use FIB-SEM to characterise mitochondrial morphology and bacterial interactions in the context of oocyte maturation and endosymbiosis.

## Introduction

Beyond their role in generating energy, mitochondria are highly dynamic organelles involved in various cellular functions^1^. Many pathogenic bacteria (e.g., *Listeria*, *Shigella*, *Rickettsia*, *Legionella,* and *Chlamydia*) are known to target and exploit the function of host mitochondria for their own benefits upon cellular invasion^2^. Multiple bacteria have been found in proximity to a host mitochondrion, such as *Chromulinavorax destructan*s in the heterotrophic flagellates *Spumella elongate*^3^ or the bacterial endosymbionts of the marine protists *Diplonema japonicum* and *Halteria geleiana*^4,5^. Intriguingly, only one bacterial endosymbiont was shown to inhabit mitochondria, namely *Candidatus* Midichloria mitochondrii (hereafter *Midichloria mitochondrii*)^6–12^.

*Midichloria mitochondrii* belongs to the order *Rickettsiales*, a group of mostly intracellular alphaproteobacteria^13,14^ known for being capable of interacting with their host cells in multiple ways^15^. Comparative genomics showed that the secretion systems and the secreted effectors of *M. mitochondrii* could play pivotal roles in mitochondrial tropism^16^. Uniquely, *M. mitochondrii* is located between the outer and the inner mitochondrial membranes of the oocytes of the European hard tick, *Ixodes ricinus* which is the main vector for Lyme disease^8,10,17^. Besides serving as an effective vector for pathogenic microorganisms, ticks form symbiotic relationships with a variety of microbes^18^. In relation to the ticks’ exclusively hematophagous diet, symbionts could provide cofactors and essential molecules^19,20^, or may be involved in blood detoxification^21^. Some tick symbionts are maternally inherited from the ovaries to offspring. These endosymbionts often establish a delicate microbial balance by precluding co-existence with other microbes or inhibiting infection by a phylogenetically related pathogen, thereby interfering with the dissemination of pathogenic strains^22,23^. Although the benefits of *M. mitochondrii* to *I. ricinus* remain unknown, the prevalence of this endobacterium in tick populations (100% in female adults and immatures) and maternal transmission indicate obligate interactions^12,24^. This hypothesis is supported by antibiotic treatment experiments, which failed to remove *M. mitochondrii*^25^. The laboratory-raised *I. ricinus*, however, appear to lose *M. mitochondrii* after several generations^24^. This line has been inbred over many generations, and it is documented to be germ-free^26,27^ as well as lacking *M. mitochondrii*^24^.

Although *M. mitochondrii* is present throughout the tick life, the highest endosymbiont concentrations were detected in blood-fed females and their eggs^9,24,28^. Blood feeding is a key event in adults of hematophagous arthropods and it initiates vitellogenisation, which is the accumulation of yolk in oocytes leading to maturation^29^. Throughout vitellogenisation, yolk granules overtake the whole cytoplasm, and the oocytes come to a metabolic standstill with stalled protein production and mitochondrial activity^30,31^. It is followed by organellar degradation, where mitochondria become smaller and spherical in different animals^31–33^. Although there is not much known regarding the involvement of mitochondria in arthropod oocyte maturation, the mammalian mitochondria and mitochondrial dynamics are known to play important roles in oogenesis^34^. Thus, the increased loads of *M. mitochondrii* upon oocyte maturation suggests a link between the oogenesis and *Midichloria* life cycle and maternal transmission.

Previously, conventional transmission electron microscopy (TEM) has enabled visualization of the intimate association between the ovarian mitochondria and *M. mitochondrii* where colonization of one to up to 15 bacteria to individual mitochondria was documented^7^. The mathematical modeling performed after quantitative image analyses of those TEM images rejected the *Bdellovibrio bacteriovorus*-like parasitism hypothesis proposed by Sacchi^7^, but instead it predicted a dynamic interaction along with the mitochondrial network^35^. However, the two-dimensional micrographs of thin-sectioned tissues are not sufficient to comprehensively display this interaction in full. In this study, we employed focused ion beam-scanning electron microscopy (FIB-SEM) to obtain label-free images of the wild-type and aposymbiotic *I. ricinus* oocytes. FIB-SEM generates a micrograph of the surface of a sample by scanning electron microscopy. Then the surface is milled by stripping the surface away using a focused ion beam, revealing the layer beneath. An image of each layer is collected, and then the entire series is aligned to yield a high-resolution 3D image of the whole sample. Here we provide the 3D images of the oocytes showing the intimate interaction between *M. mitochondrii* and mitochondria at different vitellogenisation stages. Additionally, we quantitatively describe the subcellular populations of endosymbionts and mitochondria, and accurately characterize their intersections for the first time. This advanced imaging approach reveals new symbiosis-dependent morphologies of mitochondria upon oocyte maturation with respect to the presence of the intramitochondrial symbiont.

## Results

### Two-dimensional imaging of wild-type and aposymbiotic *Ixodes ricinus* oocytes

**Fig. 1:**
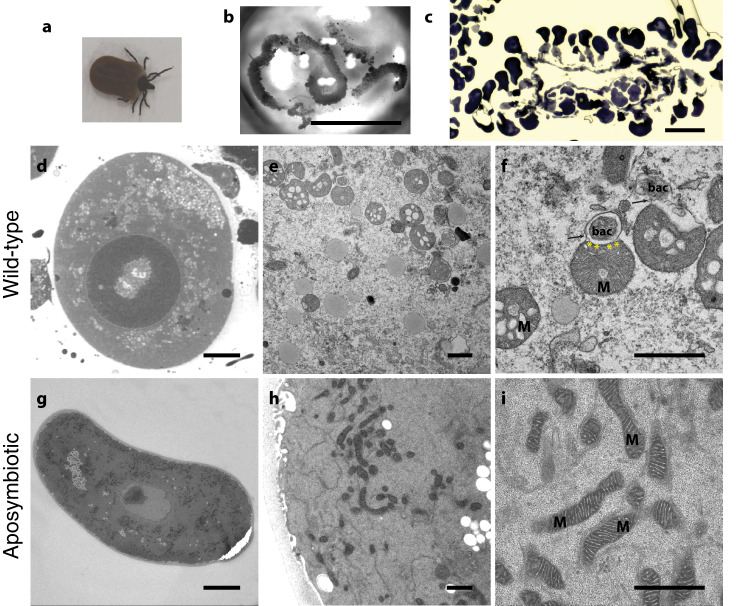
Endosymbiosis between *Ixodes ricinus* and *Midichloria mitochondrii*. **a** Adult female *I. ricinus* tick harbors *M. mitochondrii* in its **b** ovary. **c** All ovaries were investigated to visualize the multiple follicles carrying the oocytes. **d**–**f** *M. mitochondrii* is located in between the inner (yellow asterisk) and outer (arrow) membrane of mitochondria of the wild-type oocytes. **f** Conventional TEM image shows an oocyte mitochondrion (M) carrying an intramitochondrial *M. mitochondrii* (bac) underneath mitochondrial outer membrane (arrow). Note the highly vacuolated mitochondrial ultrastructure with inflated cristae. **g**–**i** Aposymbiotic oocytes have regular mitochondria with crisp cristae and are free of *M. mitochondrii*. (Scale bars **b**: 5 mm, **c**: 100 μm **d**, **g**: 10 μm; **e**, **f**, **h**, **i**: 1 μm, *n* = 1 wild-type and *n* = 1 aposymbiotic tick).

The osmium tetroxide osmium (OTO) fixation method was used for sample preparation of semi-engorged wild-type *I. ricinus* ticks (Fig. 1a) and germ-free ticks of Neuchâtel line (i.e., aposymbiotic ticks) that were dissected to harvest ovaries (Fig. 1b). The line was established from a natural population near Neuchâtel (Switzerland), and reared in the laboratory since 1978. Although controlled experiments combined with statistical data have never been reported and the generations are often lost, a drastic reduction in the prevalence of *M. mitochondrii* in younger generations was observed fifteen years ago^24^. The laboratory-raised ticks used in this work were found to be devoid of *M. mitochondrii*, while wild females were always found positive using qPCR. Since oocyte maturation is asynchronous and an ovary contains oocytes from initial development stages to the most developed form simultaneously, the epoxy-embedded ovaries were pre-examined after toluidine blue histology staining (Fig. 1c). We pre-examined both wild-type (Fig. 1d–f) and aposymbiotic oocytes (Fig. 1g–i) using conventional TEM. To visualize the mitochondria and bacteria effectively, we selected round-shaped maturating oocytes which had chorion and visible organelles but were not saturated with yolk granules. As we selected round-shaped, healthy oocytes, we did not observe any autophagic vesicles or related cellular structures. Oocytes with such structures fail to mature as healthy eggs or to be fertilized and are ultimately recycled^36^. Mitochondria of both wild-type and aposymbiotic ticks were readily visible in the high-resolution TEM micrographs due to their characteristic cristae (folds of the inner mitochondrial membrane) (Fig. 1e, f, h, i). The mitochondrial cristae of aposymbiotic ticks had a regular structure (Fig. 1h, i); however, the mitochondria of the wild-type ticks were often vacuolated (Fig. 1e, f). As expected, the electron-dense endobacteria were observed solely in the wild-type oocytes and were present in the cytosol or in the mitochondria (Fig. 1e). The intramitochondrial *M. mitochondrii* were located between the inner and the outer mitochondrial membranes (Fig. 1f), as shown previously^7^. No endobacteria were observed in the TEM micrographs of the aposymbiotic oocytes, confirming the absence of bacteria in the laboratory-reared Neuchâtel line (Fig. 1g–i).

### Simulation of a cumulative 2D imaging demonstrates the need for 3D imaging to characterize the endosymbiont-mitochondria associations

**Fig. 2:**
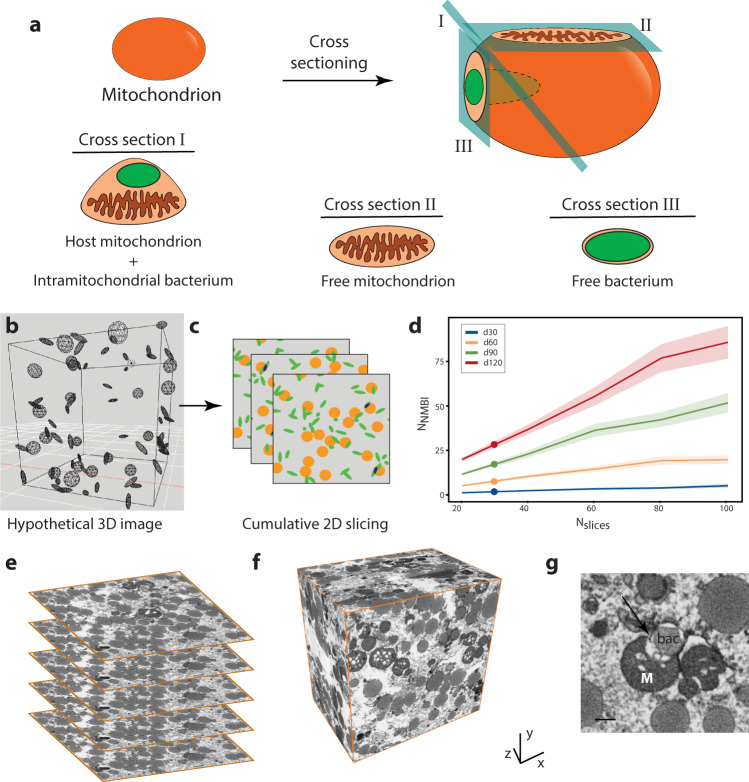
Rationale for 3D imaging and acquisition of volumetric data with FIB-SEM. **a** Schematic representation of 2D imaging of a host mitochondrion where the location of the cross-section can lead to misinterpretations (green ellipsoid: bacterium). **b** A hypothetical 3D image where mitochondria (spheres) and bacteria (ellipsoids) randomly distributed and the true number of mitochondria-bacteria intersection (NMBI) were taken. **c** For the 2D imaging simulation, multiple 2D slices were taken from the same hypothetical 3D image, and the NMBIs were calculated when bacteria (green) and mitochondria (orange) overlap (blue). **d** Graph depicting the increase in the NMBI with more 2D cross sections (*N*-slices) analyzed for object density where the centrelines show the mean and transparent bands show the 95% confidence interval for *n* = 4 object density (*d* = 30, 60, 90, 120), 90 image sets per density. The dots represent the true NMBIs calculated from 3D. **e** Consecutive electron micrographs in 10 nm distance were generated using FIB-SEM, and **f** aligned to build the volumetric images. **g** FIB-SEM imaging quality allows distinguishing typical interaction patterns throughout the 3D images; the representative image shows a mitochondrion (M) hosting a bacterium (bac) within the mitochondrial outer membrane (arrow) (Scale bar: 500 nm, *n* = 1 wild-type vitellogenic tick). Source data are provided as a Source Data file.

Although the endobacteria and the mitochondria were readily distinguishable in 2D TEM micrographs of the wild-type oocytes, a host mitochondrion can easily be misinterpreted as an endosymbiont-free mitochondrion if the plane of the section does not pass through an intramitochondrial bacterium (Fig. 2a). In order to understand whether examining a high number of TEM micrographs could adequately assess the number of mitochondria and associated bacteria in a volume, we designed an in silico study. We generated hypothetical 3D image volumes where round objects (i.e., mitochondria) and rods (i.e., bacteria) were independently distributed in random locations, and therefore sometimes intersected with each other (Fig. 2b). We generated four image sets with equal numbers of mitochondria and endobacteria, each dataset having an increasing object density (*d* = 30, 60, 90, and 120). When a bacterium and a mitochondrion overlapped, we called these cases a “mitochondrion-bacterium intersection” (MBI). We counted the true number of MBIs (NMBI) for each object density d (Fig. 2d, points represent NMBI for each d). After obtaining the true NMBI for each dataset with different object densities, we simulated 2D imaging on the same hypothetical 3D image stacks by taking random 2D slices (Fig. 2c). We took different numbers of slices (*N*-slices: 20, 40, 60, 80, 100), and calculated the total NMBIs for each slice set. As more slices were taken from a 3D stack, more intersections were observed, hence, we detected higher NMBI. The increase in the NMBI counts was directly proportional to the number of slices (Fig. 2d). Counting NMBI via cumulative 2D sectioning underestimated (Fig. 2d, *N*-slices = 20) and overestimated true NMBI (Fig. 2d, *N*-slices = 40, 60, 80, 100) depending on the number of slices. In this simulation, the closest estimate to the true NMBI was reached with *N*-slices = 30. Clearly, it is not possible to predict how many 2D images are required to correctly assess the NMBIs or how many *M. mitochondrii* reside within the mitochondria, hence, we concluded that 3D imaging was necessary to fully characterize the system.

### Three-dimensional imaging displays the cellular content of *I. ricinus* oocytes at different maturation stages

In order to document the cellular distribution of the mitochondria and bacteria and to accurately quantify free and interacting symbiotic subpopulations in 3D, we used the ZEISS Auriga Crossbeam system (Jena, Germany). FIB-SEM captures volumetric images by irreversibly removing a thin layer from the sample surface and scanning the newly revealed surface in an iterative manner that builds up to a 3D image stack (Fig. 2e, f). Due to the acquisition of backscattered electrons, FIB-SEM provides poorer resolution compared to conventional TEM (expected maximum resolution is 0.4 nm compared to 0.1 nm for TEM in biological samples)^37^. Acquiring data for a single volume takes days, allowing for drift, loss of focus, and interruption in milling to compromise the image, that further compromises the image and makes it particularly difficult to follow the mitochondrial membrane. Despite this, we obtained sufficient image resolution for detailed visualization of subcellular components of the oocytes, similar to the ones observed under conventional TEM (Fig. 1e), ensuring the details needed for thorough examination of the mitochondria and bacteria (Fig. 2g). Six acquisitions from different biological samples were processed, and we calculated the physical sizes of the imaged oocyte pieces between 346 and 6,302 μm^3^ that were products of minimum 479 to maximum 1930 slices milled on the Z axis (Table 1). Subsequently, the datasets were imported to Amira (version 2019.4) and manually segmented into separate label fields for mitochondria, bacteria, and the cytoplasm for 3D reconstruction.

**Table 1:** Details of the imaged samples

Sample	Physical size (nm)	Number of slices in Z	Image volume (μm^3^)	Cell volume (μm^3^)	Total number of mitochondria	Total number of bacteria
WT previtellogenic	10,230 × 10,230 × 11,320	1133	1185	825	79	105
WT early vitellogenic	20,470 × 20,470 × 4780	479	501	346	37	48
WT vitellogenic	20,470 × 20,470 × 19,290	1930	8083	6032	380	681
WT late vitellogenic	20,470 × 20,470 × 6970	698	3604	2839	94	197
Aposymbiotic vitellogenic	20,470 × 12,740 × 15,020	1503	3914	3284	37	0
Aposymbiotic late vitellogenic	20,470 × 20,740 × 9750	976	4085	2036	51	0

The sample names are based on vitellogenic stages of the oocytes. The physical size of the image, number of images stacked for the 3D images, the volume of the imaged sample, volume of the cell in the image, total number of mitochondria and bacteria counted in the image are listed.

**Fig. 3:**
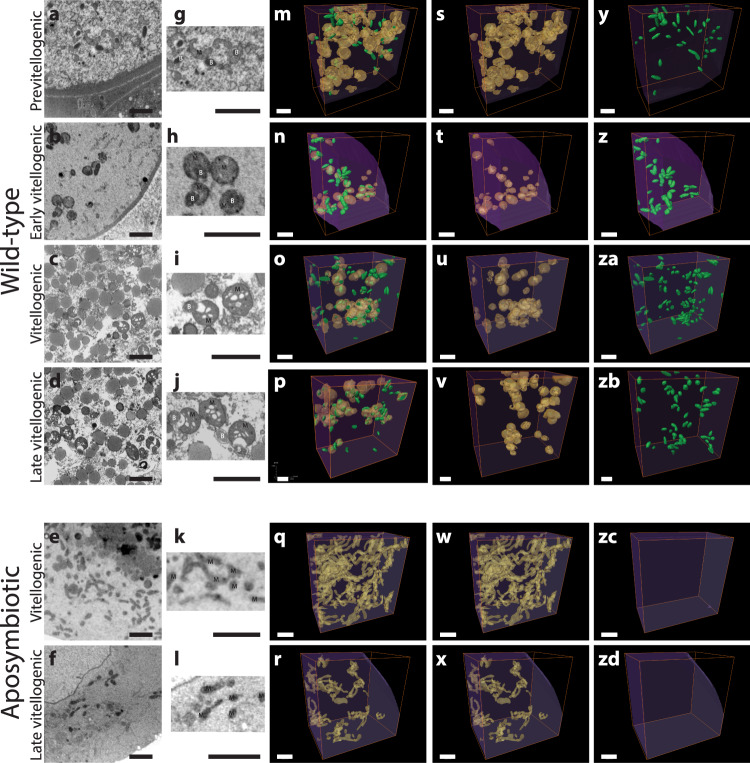
FIB-SEM generated 3D datasets of tick oocytes. The individual FIB-SEM micrographs of the wild-type (**a**–**d**) and aposymbiotic (**e**, **f**) oocytes named based on the vitellogenic stages showing the bacteria (B) and mitochondria (M). **g** The mitochondria of previtellogenic oocyte were in deformed globular shapes. As vitellogenisation began, mitochondria were globular (**h**), and remained globular as the vitellin granules accumulated (**i**, **j**). In all vitellogenic stages, intramitochondrial and cytosolic bacteria are visible (**g**–**j**). The aposymbiotic oocytes (**e**, **f**) had tubular mitochondria (**k**, **l**), forming an extensive network (**w**, **x**), without any bacteria (**zc**, **zd**). The 3D reconstructed images of equal oocytes volumes of ~50 μm^3^ show the distribution of the mitochondrial (yellow (**s**–**x**)) and bacterial (green (**y**–**zd**)) populations in the cytoplasm (purple) (**m**–**r**). (Scale bars: 2 μm *n* = 4 wild-type and *n* = 2 aposymbiotic ticks).

We examined four datasets of wild-type oocytes and two datasets of aposymbiotic oocytes from two and one semi-engorged *I. ricinus* ticks, respectively (Table 1). Similar to our 2D images, we did not observe autophagic structures or cellular damages like in the reabsorbed oocytes of arthropods. In all six datasets, we could monitor subcellular components including mitochondria and yolk particles (Fig. 3a–f). The presence of yolk particles indicated that the oocytes were under vitellogenesis. Based on the amount of yolk granules, we concluded that the oocytes were in different vitellogenic stages. Specifically, the wild-type oocytes were previtellogenic, early vitellogenic, vitellogenic and late vitellogenic (Fig. 3a–d), while the aposymbiotic oocytes were vitellogenic and late vitellogenic (Fig. 3e, f). The rod-shaped bacteria were readily distinguished and exclusively present in wild-type oocytes (Fig. 3a–d). Our investigations on bacterial appearance showed all bacteria to have electron-dense cytoplasm indicating them being healthy. Additionally, possible events of bacterial septation were detected in the oocytes cytoplasm as well as within mitochondria. However, since the resolution in FIB-SEM generated images is often compromised due to interference in the milling, we did not investigate bacterial division quantitatively.

In the previtellogenic wild-type oocyte, we could not observe any accumulated storage proteins (Fig. 3a). The cytoplasm contained intact ribosomes, vesicles, mitochondria with continuous cristae and electron-dense endosymbiotic bacteria. The mitochondria had a deformed globular shape with short extensions and *M. mitochondrii* was visible either surrounded by contortions of the extended mitochondria, or beneath the mitochondrial membrane (Fig. 3g). In the second dataset, we interpreted the aggregation of the dark particles without clear membrane as soluble vitellogenin proteins, suggesting the start of the vitellogenin transport and initiation of vitellogenesis^32^ (Fig. 3b). The cytoplasm was cleared of its contents except for vitellogenin, *M. mitochondrii* and mitochondria. We observed explicitly individual globular mitochondria without any reticulation, and some *M. mitochondrii* cells were present inside these globular mitochondria (Fig. 3h). In the third and fourth datasets, large vitellin granules were fully formed and accumulated in the cytoplasm, implying on-going vitellogenisation (Fig. 3c, “vitellogenic” and Fig. 3d “late vitellogenic”). While these vitellogenic oocytes exhibited a gradual reduction of other organelles, the mitochondria were still evenly distributed and formed individual globular structures (Fig. 3i, j). Some mitochondria still appeared intact; however, their cristae were often inflated and/or vacuolated, and occasionally the mitochondrial outer membrane was ruptured. In the images of the aposymbiotic ticks, we observed vitellin granules indicating vitellogenisation (Fig. 3e, f). Strikingly, the mitochondria formed a complex, long tubular network distinct from wild-type oocytes (Fig. 3k, l), suggesting a link between fragmentation of the mitochondrial network and presence of the endosymbiont.

### Quantitative analysis shows that a high proportion of *M. mitochondrii* are intramitochondrial

**Fig. 4:**
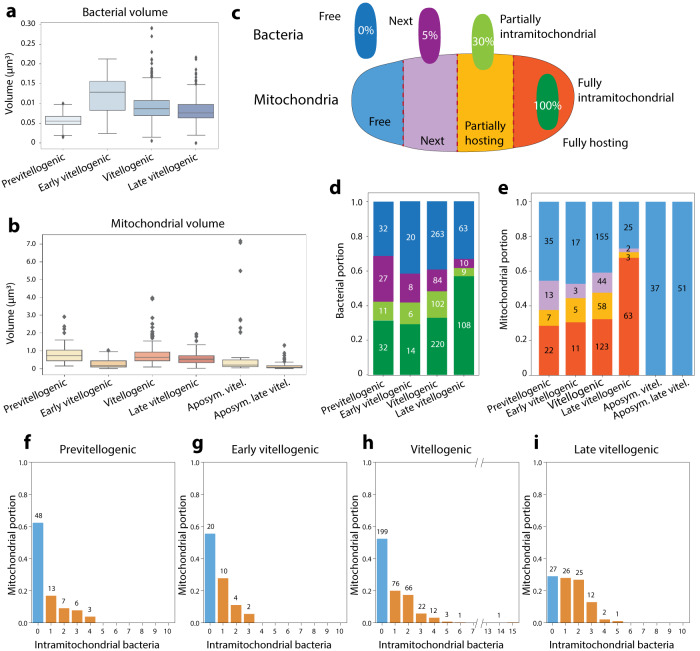
Statistical analyses of the 3D reconstructed images. Distribution of volumes of the bacteria (**a**) and the mitochondria (**b**) visualized in *n* = 4 wild-type and *n* = 2 aposymbiotic 3D datasets. In each box plot, the center line indicates the median, the edges of the box represent the first and third quartiles, and the whiskers extend to span a 1.5 interquartile range from the edges. **c** Categorization of the bacteria and mitochondria populations into subpopulations based on the volume overlap between a bacterium and corresponding mitochondrion: 0% overlap is considered a “free” bacterium, 1–5% is designated “next”, between 6 and 30% is “partially inside” and above 31 % is “fully inside”. The portions of bacterial (**d**) and mitochondrial (**e**) subpopulations in each dataset are shown. As the oocytes mature, we detected more bacteria and mitochondria in intimate association (**d**, **e**). **f**–**i** Distribution of numbers of bacteria hosted by mitochondria indicates an increase in the capacity of the mitochondrion to harbor multiple bacteria upon maturation. In the late vitellogenic stage, a mitochondrion can harbor up to 14 bacteria (**h**). Source data are provided as a Source Data file.

In all observed stages of vitellogenisation of the wild-type oocytes, mitochondria and bacteria were either (i) free in cytosol, (ii) next to a counterpart with often scarce overlap, (iii) partially or (iv) fully associated (Fig. 3m–p). We quantitatively characterized those subpopulations by computing the volumes of individual bacteria and mitochondria (Fig. 4a, b) and then calculating the Volume of Overlap Fraction (*V*_OF_ = *V*_submerged part_/*V*_bacterium_). Using V_OF_, we classified the bacteria into four subpopulations: “free” (*V*_OF_ = 0), “next” (0 < *V*_OF_ < = 0.05), “partially inside” (0.05 < *V*_OF_ < = 0.3) or “fully inside” (*V*_OF_ > 0.3), and we then grouped the partner mitochondria as “free”, “next”, “partially hosting” and “fully hosting” subpopulations based on V_OF_ of the corresponding bacteria (Fig. 4c). When there are multiple overlaps, we classified according to the largest V_OF_ (Fig. S2c).

After categorizing each bacterium (Fig. 4d) and mitochondrion (Fig. 4e) in all datasets, we found that the bacterial subpopulations “partially inside” and “fully inside” correspond to 48.4 ± 9% of the total bacteria, while 50 ± 15% of the mitochondria were found to be partially or fully hosting when the four datasets from wild-type oocytes were combined. Intriguingly, we observed that the intramitochondrial bacteria subpopulations substantially increased upon vitellogenisation (Fig. 4d). The percentage of the intramitochondrial *M. mitochondrii* increased from 42.15% in the previtellogenic oocyte to 61.57% in late vitellogenic oocyte (Fig. 4d). Moreover, the proportion of mitochondria hosting bacteria increased from 38% to 70% (Fig. 4e). In addition to this, we detected more mitochondria carrying multiple bacteria in vitellogenic and late vitellogenic oocytes (Fig. 4f–i). The portion of the mitochondrial population that harbor more than one bacterium is around 20% in the previtellogenic and early vitellogenic oocytes (Fig. 4f, g) and increased to 28% in the vitellogenic and to 43% in the late vitellogenic oocytes (Fig. 4h, i). Although the number of intramitochondrial bacteria approached five, an extreme case was observed of a mitochondrion found to host 14 bacteria (Fig. 4h). Together, our data indicate that during vitellogenisation *M. mitochondrii* increasingly associates with mitochondria, and the mitochondrial capacity to host multiple bacteria increases.

### Mitochondrial morphology changes upon the presence and number of *M. mitochondrii*

**Fig. 5:**
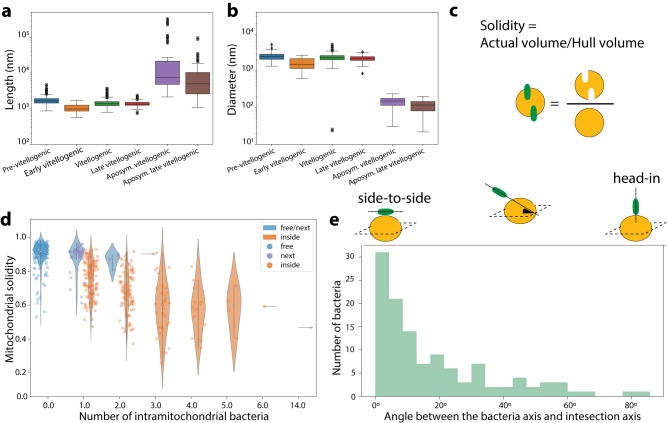
Quantitative analysis of the symbiotic partners in 3D. **a** The length and **b** diameter distributions of the mitochondria of *n* = 4 wild-type and *n* = 2 aposymbiotic oocytes. In each box plot, the center line indicates the median, the edges of the box represent the first and third quartiles, and the whiskers extend to span a 1.5 interquartile range from the edges. **c** Solidity [i.e., the ratio of the actual volume (with cavities) to the convex hull (cavities closed) volume] is taken as a descriptor of the morphologic changes in mitochondria of vitellogenic oocytes. **d** The solidity distribution of the wild-type mitochondria (*n* = 4) per number of hosted bacteria showed a decrease in solidity upon the presence and increasing number of bacteria. Violin plots, are colored according to categories indicated, and the centrelines indicate 25-75th percentiles, median, and whiskers of minima to maxima, and the width of the violin plot shows the frequency distribution of the data **e** The distribution of the angles between the mitochondrial surface and the long bacterial axis. The angle is zero when they are parallel, and approaches to 90° as bacteria become perpendicular to mitochondrial surface. Source data are provided as a Source Data file.

**Fig. 6:**
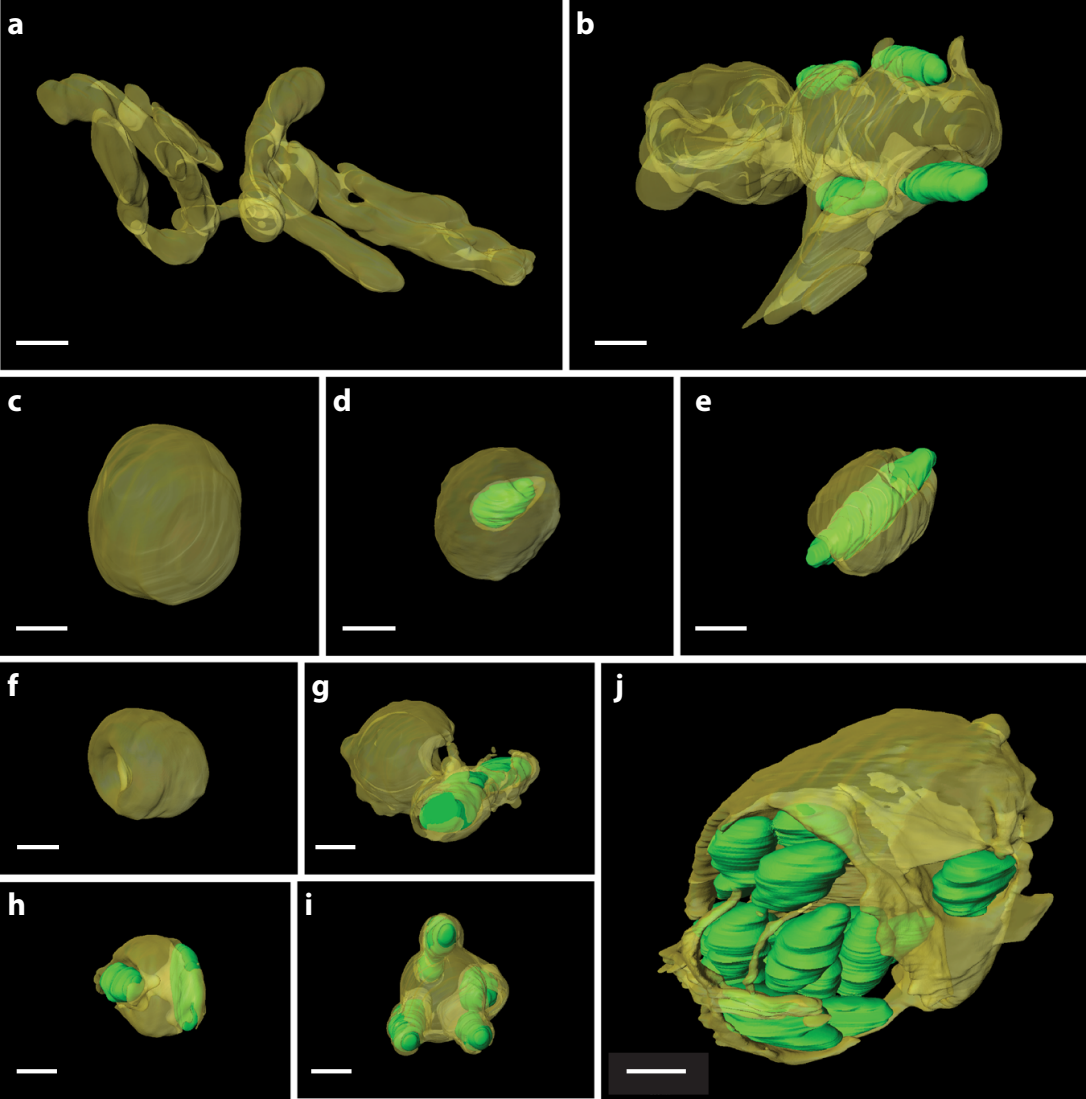
Impact of *M. mitochondrii* on mitochondrial morphology. **a** The aposymbiotic oocyte had tubular mitochondria forming a large network, whereas (**b**–**j**) no mitochondrial network is observed in wild-type oocytes. **b** The previtellogenic oocytes have a deformed globular shape with branch-like short extension. **c**–**j** All vitellogenic oocytes had globular mitochondria, some of which do not host a bacterium (**c**, **f**), host single (**d**, **e**, **g**), or multiple (**h**–**j**) bacteria. Newly described Pacman (**f**, **g**) and reservoir (**j**) phenotypes indicate pervasiveness of the intramitochondrial *M. mitochondrii*. The 3D views of each mitochondrion are presented in Supplementary Movie 1 and the slide view of a Pacman mitochondrion is shown in Supplementary Movie 2. Mitochondria are shown in transparent yellow and bacteria in green. (Scale bars: 500 nm, *n* = 4 wild-type and *n* = 2 aposymbiotic ticks).

Strikingly, we did not observe any mitochondrial network in the wild-type oocytes, while it is clearly present in aposymbiotic ones (Fig. 3s–x). In aposymbiotic ticks, lack of *M. mitochondrii* was observed simultaneously with large mitochondrial networks of tubular filaments in both vitellogenic (with diameter 140.96 ± 8.10 nm, *n* = 37 mitochondria) and late vitellogenic oocytes (diameter 106.59 ± 6.16 nm, *n* = 51 mitochondria) (Fig. 5b). The network was very long and heavily branched. It extended to 1499.69 ± 12.39 μm in the vitellogenic oocyte and 182.53 ± 1.97 μm in the late vitellogenic oocyte of aposymbiotic ticks (Fig. 5a). The longest mitochondrion reached 313.5 μm and 91.30 μm in length in vitellogenic and late vitellogenic oocytes of aposymbiotic ticks, respectively (Fig. 5a). In contrast to the large mitochondrial networks, we encountered individual, globular-shaped mitochondria of wild-type ticks with maximum axis lengths of 2.2, 0.8, 1.8 and 1.2 μm (Fig. 5a). The mitochondria of the previtellogenic-WT oocytes displayed deformed globular shapes with so-called extensions, which could not be considered as true branches (Fig. 6b). The mitochondria of three different vitellogenic oocytes had explicit globular shapes without any extensions (Fig. 6c–j). In fact, all four subpopulations of these mitochondria were globular-shaped, regardless of the presence or absence of the bacterium (Fig. 6c–j). Collectively, these data suggest that the endosymbiosis with *M. mitochondrii* has a profound impact on mitochondrial morphology.

We further examined the globular mitochondria of the wild-type vitellogenic oocytes with respect to intramitochondrial bacteria. We used solidity to quantify the disturbance in the spherical volumes^38^, defined as the ratio between the actual volume of a mitochondrion and the volume of its convex hull, which decreases when there are cavities (Fig. 5c). Twenty per cent of “free” mitochondria lacked cavities and had closed, almost spherical shapes, hence high solidities between 0.8 and 1 (Fig. 5d). The mitochondrial subpopulation “next” also had high solidity scores. The presence of a bacterium in the mitochondria led to a decrease in solidity down to 0.24, and 23% of the host mitochondria had solidities between 0.8 to 1. Having more bacteria in a mitochondrion formed more cavities; hence the solidity decreased more (Fig. 5d). This quantitative and unbiased 3D analysis is in line with the microscopic observations in which many mitochondria are vacuolated and inflated, and occasionally the outer membrane is ruptured. Overall, these results show that mitochondria disfigure in the presence of multiple intramitochondrial *M. mitochondrii*.

### *Midichloria mitochondrii* lacks spatial arrangement in the cytosol

Aside from the intramitochondrial bacteria, the orientation of the cytoplasmic *M. mitochondrii* relative to the closest mitochondria drew our attention in terms of their inclination toward mitochondria. We investigated whether the bacterial subpopulation “next” exhibits a preferred orientation with respect to the overall structure of corresponding mitochondria by measuring the angle between mitochondrion and bacterium axes (Fig. 5e and S3). We obtained 103 angles between the “next” bacteria and their corresponding mitochondria and observed a bias towards smaller angles (<20°). This suggests a preferred “side-to-side” orientation over the “head-in” orientation (Fig. 5e). As rod-shaped bacteria prefer polar motion, our observations suggest that *M. mitochondrii* does not target the mitochondria along the polar axis.

### Symbiosis-dependent mitochondrial morphologies

By monitoring the mitochondrial membrane in full and showing how completely it surrounds the intramitochondrial bacterium, new morphologies of the mitochondria in *I. ricinus* oocytes were observed (Fig. 6, and their 3D representations in Supplementary Movie 1). In all screened stages of oocyte vitellogenisation, we detected three notable phenotypes of mitochondria: the mitochondrial network present in aposymbiotic oocytes (Fig. 6a), the previtellogenic deformed globular shape with short extensions (Fig. 6b), and the globular mitochondria of the wild-type oocytes (Fig. 6c–j). In most cases, one bacterium was hosted by one mitochondrion (Fig. 6d, e, g). Once the *M. mitochondrii* was inside the mitochondria, it tended to be closer to the outer mitochondrial membrane and stand apart from the mitochondrial matrix (Fig. 6b, h, i), yet we observed a few examples where the bacterium was deeper inside the mitochondrion (Fig. 6d, e, j). Furthermore, we found that bacteria could pass through the mitochondria along their long axes, occasionally exposing one or both bacterial poles (Fig. 6e). The typical globular shape and mitochondrial membrane were maintained intact in most of those mitochondria (Fig. 6d, e). Remarkably, we observed several examples where a completely intact outer mitochondrial membrane fully enclosed a bacterium, providing compelling evidence for complete internalization of *M. mitochondrii* (Fig. 6d).

In some cases, we observed a separation of the outer and inner membrane, sometimes in a largely inflated mitochondrial intermembrane space (Fig. 6f) and occasionally distorted mitochondria with distant bacteria (Fig. 6g). These mitochondria often had ruptured membranes and split components, and the intramitochondrial bacterium remained surrounded by mitochondrial remnants (Fig. 6g, Supplementary Movie 2). We termed these mitochondria as “Pacman mitochondria” due to their major spherical sector shape. Interestingly, Pacman mitochondria could be free (Fig. 6f) or host a bacterium (Fig. 6g, Supplementary Movie 2) and were exclusively present in the vitellogenic and late vitellogenic wild-type oocytes. We observed no distinct mitochondrial membrane around the cavity of the free Pacman mitochondria (Fig. 6f). Aside from the cavity in free Pacman mitochondria, the rest remained intact with solid cristae. Intriguingly, when a bacterium was inside a Pacman mitochondrion, the mitochondrial membrane outwardly ruptured, rather than being curved inward (Fig. 6g, Supplementary Movie 1, Supplementary Movie 2).

Many mitochondria of wild-type oocytes, especially those in late vitellogenesis, were detected to host multiple bacteria, and they maintained their globular shape and intact matrix (Fig. 6h–j). The cavities containing the bacteria were partly (Fig. 6h) or completely (Fig. 6i) enclosed by the mitochondrial membranes, and the bacteria would lie along the membrane. In the vitellogenic oocyte, we observed one exceptional mitochondrion with enlarged volume and surface area with a solidity score 0.47 (Fig. 6j). It had a significantly increased convex hull volume (9.5 μm^3^) compared to the average mitochondrial volume (1.1 μm^3^) and displayed a large void interior of 5.3 μm^3^. Strikingly, this host mitochondrion is not only the largest detected but also contained the highest number of bacteria. Fourteen bacteria were found in this over-colonized mitochondrion that resembles a bacterial “reservoir” in which *M. mitochondrii* is fostered (Fig. 6j, Supplementary Movie 1j). The bacteria were observed to line up against the membrane or occupy the interior of the reservoir mitochondrion, leading to significant enlargement of the cavity (Fig. 6j, Supplementary Movie 1j). An excessive number of bacteria in a single mitochondrion might have caused inflation of the mitochondrion followed by rupture of the membrane, which could release the bacteria. The lack of autophagic structures in the vicinity of the reservoir mitochondria further rules out mitochondrial response to such overpopulation. Together with the spatial arrangements of the “next” bacterial subpopulations, the Pacman mitochondria where bacteria seem to depart the mitochondria, and, lastly, the reservoir mitochondria with multiple bacteria and damaged cristae, our data confirm that *M. mitochondrii* preferentially lives intramitochondrially. Mitochondria appear to mostly associate with the bacteria, while no evidence was observed for re-colonization once the bacterium is in the cytosol. Hence, the intramitochondrial *M. mitochondrii* proliferation and rupture of the host mitochondrial membrane might give rise to the cytosolic subpopulation of the endosymbiont.

## Discussion

The obligate endosymbiont *M. mitochondrii* is located in the mitochondria and the cytoplasm of the oocytes of the European hard tick, *I. ricinus*. Although previous high-resolution micrographs showed that *M. mitochondrii* resides in the cytosol and in the intermembrane space of oocyte mitochondria, acquiring comprehensive images of specific subcellular interaction has been constrained by their relative geometry and the angle through which they were cross-sectioned. Conventional TEM images can be misleading depending on the position of the cross-section and having multiple images might not help to understand the overall interaction. In fact, our simulations showed that cumulative 2D cross-sectioning cannot adequately assess the intersecting objects in hypothetical 3D volumes. As more hypothetical cross sections were screened, the NMBIs unpredictably exceeded the true values of the hypothetical 3D images, likely due to counting the intersections repetitively. We concluded that 2D imaging of tick oocytes is not sufficient to accurately count the subcellular populations in oocytes or to display the bacterial-mitochondrial intersection in its entirety and resorted to 3D imaging.

In this study, we used FIB-SEM to obtain large 3D images displaying the complete symbiotic interface, demonstrated the interacting partners in full, and quantitatively characterized the subpopulations at oocyte level. Our FIB-SEM micrographs of six datasets had sufficient image resolution to confirm the details provided in TEM images in which we could observe the interaction between mitochondria and *M. mitochondrii* at different vitellogenic stages. Vitellogenisation in both wild-type and aposymbiotic oocytes was associated with organellar degradation and clearance of cytoplasm, while mitochondria remained unaffected by the decay. In aposymbiotic oocytes, mitochondria contained intact continuous membranes, and healthy cristae in vitellogenic and late vitellogenic stages. In contrast, wild-type mitochondria had inflated cristae, and the vacuolation was more prominent in later vitellogenesis. Such mitochondrial appearance in wild-type oocyte was independent of the presence of intramitochondrial *M. mitochondrii* but related to oocyte maturation. Additionally, *M. mitochondrii* is detected in previtellogenic stage, and associated with mitochondria, which means the endosymbiont is already in the oocyte and intimately associated with mitochondria before vitellogenisation.

The 3D reconstruction and quantitative analyses showed that, in general, approximately half of the bacterial and mitochondrial populations in the oocytes were associated with a symbiotic partner. We observed an increase in the portion of both intramitochondrial bacteria and host mitochondria during vitellogenisation. In addition, a substantial increase in mitochondrial capacity to host multiple bacteria was detected. Increasing frequency of interacting partners upon vitellogenesis show that the intimate association is favored during oocyte maturation. Previously, highest amount of *M. mitochondrii* was detected after blood feeding and oviposition using qPCR^9^ which could be linked to the increase in the intramitochondrial subpopulation.

Our 3D images displayed the mitochondria in ultrastructural detail and provided novel insights. The striking observation was the contrast between the presence of a mitochondrial network in aposymbiotic oocytes and the absence of a network in wild-type. The high-resolution 3D images revealed that an extensive mitochondrial network was only present in aposymbiotic oocytes. It would have been desirable to observe the mitochondrial dynamics using fluorescent microscopy; however, live imaging was not possible as the oocyte mitochondria are presumably inactive, similar to mammalian oocytes^30,39^. Also, the hydrophobic chorion and the excessive lipoprotein content of the oocytes severely hampered any tick-specific genetic or immunohistochemical approaches without compromising the spatial resolution. Still, the FIB-SEM snapshots at different vitellogenic stages did provide information on changes in mitochondrial morphologies upon oogenesis. The wild-type previtellogenic oocytes had network-like, deformed globular mitochondria, whereas the mitochondria of vitellogenic oocytes had solely globular shapes without any reticulation. Similarly, the elongated tubular mitochondria forming an intricate network in the aposymbiotic oocytes was more reticulated in the late vitellogenic stage. This indicates mitochondrial fragmentation upon vitellogenisation in both wild-type and aposymbiotic oocytes. In fact, the mitochondria of many mammalian oocytes have been observed mainly as spherically shaped organelles with vacuolations and few cristae^40^. Also, the TEM images of hard ticks *Amblyomma cajennense*^41^, *Rhipicephalus microplus*^36^, *Rhipicephalus sanguineus*^32^, *Amblyomma triste*^42^ showed rounded mitochondria, often with vacuolations, suggesting that the phenomenon is widespread in the Ixodidae family. In our study, the microscopic snapshots point to mitochondrial fission during oogenesis, possibly to deliver healthy mitochondria to the offspring. The contrast in mitochondrial morphologies we observed might indicate an impaired mitochondrial fragmentation in aposymbiotic ticks due to the absence of endosymbiont. The manipulation of mitochondrial dynamics by the symbiont seems to be a parasitic behavior, however an overall beneficial effect of the interaction on host fitness is suggested by the 100% prevalence of *M. mitochondrii* in *I. ricinus* populations^24^, and potential B-group vitamin supply to the host^12,43^. Indeed, the endosymbionts in evolutionary transition from pathogen to mutualist are known to retain pathogenic features in order to manipulate hosts for their survival^44,45^. In the case of *M. mitochondrii* and the oocyte mitochondria, the mitochondrial fragmentation upon oogenesis appeared to be manipulated by the endosymbiont for its transmission. The stalled mitochondrial activity in oogenesis could be sensed by the ATP/ADP translocase whose gene is encoded on the *M. mitochondrii* genome^12^ and can act as a signal for host manipulation. This could further explain the difficulty to obtain and maintain a *M. mitochondrii*-free tick line and the obligatory nature of the interaction.

The symbiosis-dependent mitochondrial phenotypes highlight the dynamic interaction between the endosymbiont and the mitochondria. Accommodation of the intramitochondrial bacterium not only creates bacterial cavities inside the mitochondria but also leads to considerable shrinkage of the mitochondrial matrix and a greatly inflated intermembrane space, which we measured as a decrease in solidity. Those cavities were often enclosed by the mitochondrial membrane, yet the membrane was occasionally ruptured, especially in Pacman mitochondria where the membrane ruptured outwardly. In the particular examples (Fig. 6g, Supplementary Movie 1g), the shapes appear to be caused by a former intramitochondrial, currently cytoplasmic, bacterium that applied forces to vacate the mitochondrion and rupture mitochondrial membrane outwardly. In fact, the appearance of the free Pacman mitochondrion leads to speculations that the cavity is formed after a former intramitochondrial bacterium abandoned its host. Furthermore, the orientation of cytoplasmic *M. mitochondrii* towards the closest mitochondrion could further highlight the dynamics of the interaction. The bacteria in close proximity to mitochondria (i.e., subpopulation “next”) do not appear to poke the mitochondria with their poles, but rather stand side-by-side and seem unresponsive to the closest mitochondrion. We did not obtain any evidence of cytosolic bacteria invading the mitochondria, and we also observed mitochondrial membrane surrounding the bacterium without being poked or ruptured in an inwardly fashion, which suggests an exit. On the other hand, we detected the heavily colonized reservoir mitochondrion harboring 14 bacteria without major destruction in its morphology. Previously, the reservoir mitochondria were detected in TEM images that led to the proposal of the *Bdellovibrio bacteriovorus*-like parasitism hypothesis and suggested patterns of destruction of the mitochondria^7^. However, the modeling of quantitative 2D microscopy data on *I. ricinus* oocytes, while confirming these highly colonized mitochondria to be rare but constantly present, has already ruled out the *B. bacteriovorus*-like parasitism hypothesis and suggested dynamic interactions of the bacteria with the mitochondrial network^35^. Our 3D images of the reservoir mitochondrion in its entirety showed that, despite the ruptured membrane, the cristae of the reservoir mitochondrion were still intact, and no marks of mitophagy were detected. In fact, the phenotype could be the result of a mitochondrial enhancement of bacterial proliferation, such that *M. mitochondrii* is persistent throughout *I. ricinus* life stages.

In this study, we presented 3D images of the *I. ricinus* oocytes, displaying the symbiotic interaction in different vitellogenic stages and quantitative information in populations. Beyond displaying the entirety of mitochondria and bacteria in 3D and demonstrating the full inclusion of *M. mitochondrii* inside a mitochondrion for the first time, we described the symbiosis-dependent morphologies. Together with the orientation of the cytosolic bacterial populations, our findings point out that *M. mitochondrii* thrives in an intramitochondrial lifestyle fostered by the mitochondria, and vitellogenisation is associated with substantial increases the intramitochondrial bacterial population and host mitochondria. The presence of a mitochondrial network solely in aposymbiotic oocytes suggests an endosymbiont-regulated mitochondrial fragmentation that is required for successful oogenesis. By providing quantitative information on subcellular populations in *I. ricinus* oocytes and phenotypic characterizations of the symbiosis-dependent mitochondrial morphologies, our 3D images could inform further molecular studies that will deepen the understanding of mitochondrial tropism. With the possibility of targeted-imaging of mitochondrial dynamics and the endosymbiont, additional studies could display the major players in endosymbiont-mediated mitochondrial fragmentation in oogenesis. Such quantitative evaluation will further help in understanding the persistence and maternal transmission of the endosymbiont.

## Methods

### Ethics statement

All mammalian handlings were carried out in accordance with the European Community Council Directive of September 22, 2010 (2010/63/EU). Four semi-engorged *I. ricinus* ticks were collected from goats in Northern Italy in September 2018 by an authorized veterinarian and naturally attached wild ticks were harvested in the context of routine sanitary screenings. The procedures were in accordance with current Italian law on the use of animals in science. Four adult female Neuchâtel ticks were reared and engorged on rabbits in the BIOEPAR Laboratory, Nantes, France (agreement number E44271). The project APAFIS#19700-20190309v1 (Engorgement of *Ixodes ricinus* ticks on rabbits) was authorized by the CEEA-06 (Comité d’Ethique en Expérimentation Animale Pays de la Loire) in April 2019. Rabbit was housed in breeding cages at constant temperature (22 ± 1 °C) and relative humidity (50%), with a 12:12 h light:dark cycle (light on 07.00–19.00 h). Food and water were available *ad libitum*.

### Tick sampling

Ticks were delivered to Institut Pasteur, France in ambient temperature in 50% humidity following the biosafety protocols of European Parliament. They were kept in ambient temperature in 85% humidifying chamber until dissection. The dissection was performed manually under an Olympus SZ60 (Japan) stereo microscope, and the ovaries were collected. Tick photographs were taken using a Canon EOS 1100D (Japan) digital camera. The presence of *M. mitochondrii* was determined via *gyrB* specific qPCR (forward: 5′-CTTGAGAGCAGAACCACCTA-3′ and reverse: 5′-CAAGCTCTGCCGAAATATCTT-3′, amplifying 125 bp) as previously described^9^, and the Neuchâtel line did not show any amplification.

### Sample preparation for electron microscopy

We have used the OTO staining method which is suitable for effective penetration of fixative and contrasting agents into tick ovaries to be imaged using FIB-SEM^37^. The harvested ovaries were washed 3 times in PBS and fixed in 2.5% glutaraldehyde, 4% paraformaldehyde (Electron Microscopy Sciences, MA, USA) in 0.2 M cacodylate buffer (pH 7.2) overnight at 4 °C. Fixed samples were washed three times by the addition of fresh 0.1 M cacodylate buffer (pH 7.2) and post-fixed in 1% osmium tetroxide (Electron Microscopy Sciences) in 0.1 M cacodylate buffer enriched with 1.5% potassium ferrocyanide for 1 h in the dark. After three washes in water, the samples were incubated for 30 min at room temperature in 0.2% of tannic acid in water. Samples were post-fixed a second time in 2% osmium for 1 h, washed in water and incubated overnight at 4 °C in 1% uranyl acetate in ethanol 25%. Samples were gradually dehydrated in an ethanol series from 50% to 100% and then embedded in epoxy resin (PolyBed812) (Electron Microscopy Sciences), followed by polymerization for 48 h at 60 °C. All chemicals were from Sigma-Aldrich (Saint Quentin Fallavier, France), unless otherwise stated.

### Transmission electron microscopy

Semi-thin sections (500 nm) obtained from each block were stained with toluidine blue to find the region of interest before cutting thin sections in 70 nm using an ultramicrotome (Leica EM UC7, Vienna, Austria). The sections were post-stained with aqueous solutions of 4% uranyl acetate for 40 min and 3% lead citrate for 10 min. The TEM micrographs were acquired using a FEI Tecnai T12 120 kV (Thermo Fisher Scientific, Les Ullis, France). The micrographs in Fig. 1 were edited in Fiji for adjustment of brightness, contrast and blurriness. The micrographs in Fig. 1g was edited in Adobe Photoshop version 20.0.7 to remove the background for display purposes.

### Focused ion beam-scanning electron microscopy

For FIB-SEM image acquisition, the resin-embedded samples were mounted on aluminum stubs, with the pyramidal surface of the resin block pointing upwards. The block surface was sputter-coated with a 20 nm thick layer gold-palladium in a Gatan Ion Beam Coater 681 (Gatan, CA, USA) and an additional 2 nm platinum layer placed using the gas injection system inside the Auriga Crossbeam Field Emission Scanning Electron Microscope (Carl Zeiss, Jena, Germany) workstation microscope chamber. The specimen stage was tilted at 54° with 5 mm working distance from the pole piece, at the coincidence point of the electron and the gallium beams. The milling conditions for the trench that allowed the view of the cross-section were 10 nA at an accelerating voltage of 30 kV. The fine polishing of the surface block was performed with 5 nA at 30 kV. For the slice series, 1 nA milling current was applied, removing a 10 nm layer from the specimen block surface. Scanning EM images were recorded with an aperture of 60 μm in the high-current mode at 1.5 kV of the in-lens EsB detector with the EsB grid set to −300 to −500 V. The voxel size is 10 nm in *x*, y, and *z*. The contrast of backscattered electron images was inverted and acquired using ATLAS 5 software (Carl Zeiss, Germany). Acquisition time of each volume lasted on average 3 days, and was sometimes accompanied by drift, loss of focus, and interruption in milling, resulting in volumes of different sizes. We acquired 2048 nm × 2048 nm images and continued until milling was interrupted. These were aligned and processed using Fiji^46^ plugin Stacks—shuffling/Align Slices^47^.

### Three-dimensional-structure reconstruction and volume analysis

Image series obtained using ATLAS 5 software were transferred to ImageJ v.1.52t for alignment and reconstruction of 3D images. The 3D images were analyzed using 3D visualization software Amira version.2019.4 (Thermo Scientific, USA). The mitochondria, bacteria and cell membrane were manually segmented, and the volume of representative data was measured using Amira. For the analysis in Fig. 5, the volumes of total mitochondria in a unit cubic volume (1 µm^3^) were measured using the same software. The methodology of simulations, 3D image categorization and the analyses are explained in details in supplementary material. The graphical representations of the 3D images were generated using ImageJ v.1.52t, Amira version 2019.4 and Adobe Illustrator v.26.0.1. The rotational and slide view of the 3D reconstructions were obtained using Amira version 2019.4 and supplementary movies 1 and 2 were prepared using Adobe Premiere Pro v.13.1.5.

### Reporting summary

Further information on research design is available in the Nature Portfolio Reporting Summary linked to this article.

## Supplementary information

Supplementary Information

Peer Review File

Description of Additional Supplementary Files

Supplementary Movie 1

Supplementary Movie 2

Reporting Summary

## Data Availability

The 3D images and the image reconstructions generated and analyzed during this study are not publicly available due to size constraints, but they are available from the corresponding author on reasonable request. Exemplary electron micrographs and 3D reconstructions are represented in Fig. 3. Source data for the statistical analyses are provided in this paper. Source data are provided in this paper.

## Source data

Source Data

## Code availability

The simulations are produced using the open-source software as described in the supplementary material. Source data for the simulation outcome are provided in this paper.
