# Supplementary material for: Three-dimensional images reveal the impact of the endosymbiont Midichloria mitochondrii on the host mitochondria
Source: Nat Commun. 2023 Jul 12;14:4133. doi: 10.1038/s41467-023-39758-x (PMC10338478; doi:10.1038/s41467-023-39758-x)
Supplement: Supplementary file 1 — Supplementary Information [file 41467_2023_39758_MOESM1_ESM.pdf]

Supplementary Material for:

Three-dimensional images reveal the impact of the endosymbiont *Midichloria mitochondrii* on the host mitochondria

Zerrin Uzum[a,*], Dmitry Ershov[b,c], Michael J. Pavia[d], Adeline Mallet[d], Olivier Gorgette[d], Olivier Plantard[e], Davide Sassera[f], Fabrizia Stavru[a]

[*] To whom correspondence should be addressed: zerrin.uzum@asu.edu

Table of Contents

1. Simulation of cumulative 2D imaging

2. Image analysis

a) Categorization of bacterial and mitochondrial subpopulations based on volume overlaps

b) Angle between bacteria and mitochondria

c) Mitochondrial length measurement

3. References

This file includes:

Figures S1, S2 and S3

Simulation of cumulative 2D imaging

To produce simulated 3D mitochondria-bacteria ensembles in a cube-shaped volume, a fixed number of solid bodies representing mitochondria (spheres) and bacteria (ellipsoids) first populate the faces of the cube randomly (uniform distribution on faces) in the beginning of the simulation. These bodies obey the following interaction rules: bacteria cannot intersect each other, mitochondria cannot intersect each other, and bacteria may intersect with mitochondria. At the onset of the simulation, these bodies were emitted from the faces of the cube inwards (normal to the faces) and allowed to collide for 100 frames. Out of these 100 frames, we discarded the 10 first frames because the solid bodies were still too close to the faces they had been emitted from. Thus, each time-lapse provides 90 unique 3D mitochondria-bacteria ensembles.

To account for the effect of object density, we varied the density of mitochondria and bacteria d (dmito = dbact = d) and generated 4 time-lapses: d=30, d=60, d=90 and d=120, and each frame in time lapses was converted into label field data. Thus, for each d we had 90 unique sets of volumetric data, from which we counted the number of mitochondria-bacteria intersections (NMBI) in 3D. For any d, we observed no global trend of NMBI in time (Fig. S1b): there was only stochastic fluctuation of NMBI around a mean value that grew with the number of objects N (Fig. S1a).

After obtaining the true NMBI values, we counted NMBIs using 2D sampling, by taking a number of 2D slices from a 3D volumetric data. We randomly sampled several 2D slices (N_{slices}) from a 3D volume and summed all NMBIs detected in all of the slices. In Fig. 3 we show the

effect of N_{slices} on N_{MBI}, calculated using this approach. Similar to 3D analysis, there was no time effect, and the number of intersections grows with the number of samples slices.

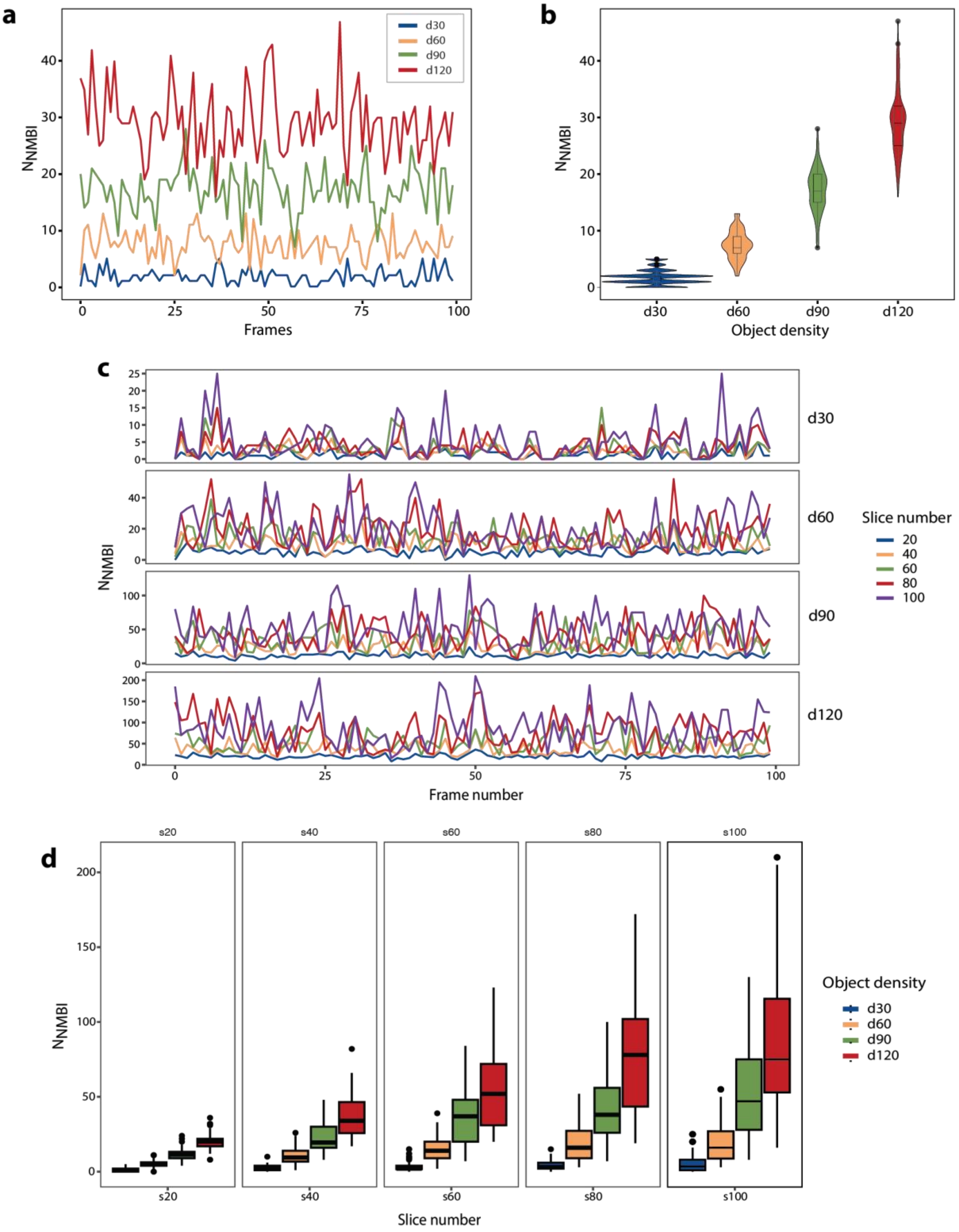

Figure S1: Effect of object number N on the number of mitochondria-bacteria intersections (NMBI) in 4 sets of 90 unique simulated 3D image stacks (*n* = 360 stacks). **a** Graph depicting the number of NMBI detected in imaging sets; there are only stochastic fluctuations around expected values. **b** Distribution of true NMBI for each set of N. **c** Effect of the number of sampled slices (Nslices) on the number of mitochondria-bacteria intersections, N_{MBI}. (A-D): each panel shows different object density: d=30, 60, 90 and 120. **d** Effect of the number of samples slices (Nslices) on the number of mitochondria-bacteria intersections, NMBI. In each box plot, the center line indicates the median, the edges of the box represent the first and third quartiles, and the whiskers extend to span a 1.5 interquartile range from the edges.

Image analysis

Exporting Amira segmentation as labelled images was done in FIJI / ImageJ 1.52p running in JDK 1.8 environment. Processing, analysis and results plotting was done in Python environment: Python 3.6.6, skimage 0.14.2, matplotlib 2.2.2, seaborn 0.9.0, numpy 1.16.4, scipy 1.3.1, pandas 0.25.1. We used IDE (Integrated Development Environment) of Jupyter: jupyter core 4.5.0, jupyter-notebook 6.0.1, qtconsole 4.5.1, ipython 7.8.0, ipykernel 5.1.2, jupyter client 5.3.1, jupyter lab 1.1.4, nbconvert 5.5.0, ipywidgets 7.5.1, nbformat 4.4.0, traitlets 4.3.

a) Categorization of bacterial and mitochondrial subpopulations based on volume overlaps

In order to evaluate subpopulations of the mitochondria and the bacteria and their spatial relationship, we exported the 3D reconstruction information from Amira obtained 3D segmentations as standard label images (labelled connected components/mitochondria and bacteria) for further processing and analysis in Python environment, then we categorized bacteria and mitochondria based on the volume of their overlaps (label intersections) after

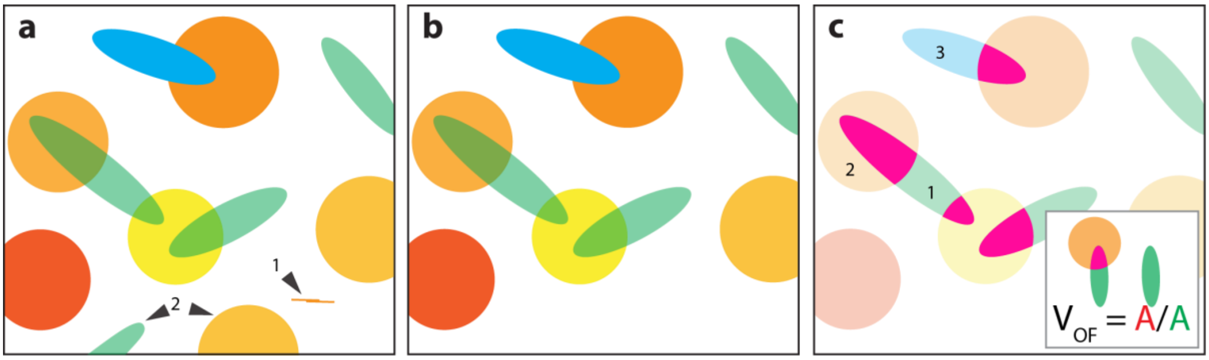

Figure S2. Calculation of Volume overlap fraction V_{OF}. **a** Original label image (2D representation) with junk pixel clusters (1) and objects touching the border (2). **b** Cleaned label image. **c** Overlaps are shown in red; V_{OF} is calculated as ratio of the overlapping volume to the total volume of a bacterium.

First, we remove unreliable objects (Fig. S2A): detached pixel clusters (arrow 1), objects touching the image borders, if the volume of such a label was smaller than 75% of the expected (mean) volume (*e.g.* if the bacteria typical volume is 100 pixels and the volume of a bacterium label touching a border of a 3D stack is below 75 pixels, then this label is removed from analysis, Fig. S2a arrows 2). This produces a clean label image ready for further analysis (Fig. S2b). Second, we find all existing overlaps between individual bacteria and mitochondria (Fig. S2c, purple regions). We used the convex hull volume of mitochondria to check for overlaps.

We observed the following types of overlaps: a single bacterium may overlap with none, one, or several distinct mitochondria; the same for mitochondria: a single mitochondrion may overlap with none, one or several distinct bacteria. If a bacterium overlaps with more than one mitochondrion, we determine one "significant" hosting mitochondrion: the one with the largest volume overlap (Fig. S2c, bacterium 1, mitochondrion 2). If a bacterium overlaps with one mitochondrion (Fig. S2c, bacterium 3), there is only one "significant" host. For each

bacterium, we calculate volume overlap fraction V_{OF} as the ratio between the volume of overlap and the full volume (Fig. S2c, inset).

Then we categorize bacteria into 4 groups according to their V_{OF}: “free” ($V_{OF} = 0$), “next” ($0 < V_{OF} <= 0.05$), “partially inside” ($0.05 < V_{OF} <= 0.3$) and “fully inside” ($V_{OF} > 0.3$). We categorize mitochondria into the same groups based on V_{OF} of the hosted bacteria; if there were several intramitochondrial bacteria, we assigned the category of the most extensive overlap (*e.g.* if a mitochondrion hosts “next” and “partially inside” bacteria, then this mitochondrion is categorized as “partially inside”).

b) Angle between bacteria and mitochondria

To calculate the angle between a “next” bacterium and its associated mitochondrion, we use the standard definition of the angle between intersecting line and plane. In our case, the line will be the longest axis of a bacterium (Fig. S3, solid green lines) and the plane will be the intersection of the bacterium and the convex hull of its hosting mitochondrion (Fig. S3, dashed blue lines). Typically, the shape of such intersection in 3D resembles a flat or slightly bent disk, which we fit with a plane using PCA method (Fig. S3c). We define the angle between the mitochondria surface and the bacterium alpha as the angle between the intersection plane and the bacterium longest axis; thus, if the bacterium longest axis is normal to the intersection plane, then the angle takes values between 0 (scenario 1 in Fig. S3a) and 90 (scenario 3 in Fig. S3a).

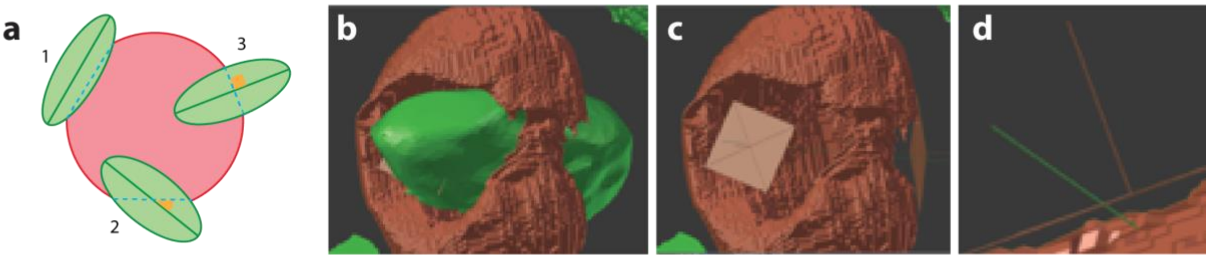

Figure S3. Calculation of the angle between a bacterium and its hosting mitochondrion. **a** Mitochondrion convex hull (pink sphere) and its intersection (blue dashed lines) with a hosted bacterium (green ellipse); the angle of interest is indicated with orange patches. Three example scenarios are shown: (1) Parallel orientation, angle = 0; (2) angle = 45; (3) Perpendicular orientation, angle = 90. **b** An example of intersecting in 3D. **c** There are two intersection planes; the frontal one is clearly visible, and the rear can be seen behind the mitochondrion. **d** Zoom-in on the frontal intersection plane; green line indicates the bacteria longest axis, and the red line is the normal to the intersection plane.

c) Mitochondrial length measurement

The length of mitochondria in the aposymbiotic oocyte was calculated with Centreline module of Amira version 2019.4. To measure the length of the globular mitochondria of the wild-type oocytes, we used the longest axis of fitted 3D ellipsoids; this method proved to be the most robust in ignoring the bacteria-associated cavities present inside mitochondria (in contrast to skeletonization and centre line). The fitting was done using a FIJI plugin “3D suite”[1].

References:

1 Ollion, J., Cochennec, J., Loll, F., Escudé, C. & Boudier, T. TANGO: a generic tool for high-throughput 3D image analysis for studying nuclear organization. Bioinformatics **29**, 1840-1841, doi:10.1093/bioinformatics/btt276 (2013).
