# Supplementary material for: Three-dimensional images reveal the impact of the endosymbiont Midichloria mitochondrii on the host mitochondria
Source: Nat Commun. 2023 Jul 12;14:4133. doi: 10.1038/s41467-023-39758-x (PMC10338478; doi:10.1038/s41467-023-39758-x)
Supplement: Supplementary file 3 — Description of Additional Supplementary Files [file 41467_2023_39758_MOESM3_ESM.pdf]

Description of Additional Supplementary Files

File Name: Supplementary Movie 1
Description: The 3D views of each mitochondrion presented in figure 6 (scale bar: 500 nm).

File Name: Supplementary Movie 2
Description: The 3D the slide view of a Pacman mitochondrion (scale bar: 500 nm).
