# Supplementary material for: Three-dimensional images reveal the impact of the endosymbiont Midichloria mitochondrii on the host mitochondria
Source: Nat Commun. 2023 Jul 12;14:4133. doi: 10.1038/s41467-023-39758-x (PMC10338478; doi:10.1038/s41467-023-39758-x)
Supplement: Supplementary file 6 — Reporting Summary [file 41467_2023_39758_MOESM6_ESM.pdf]

nature portfolio

Corresponding author(s): Zerrin Uzum

Last updated by author(s): Jun 7, 2023

# Reporting Summary

Nature Portfolio wishes to improve the reproducibility of the work that we publish. This form provides structure for consistency and transparency in reporting. For further information on Nature Portfolio policies, see our Editorial Policies and the Editorial Policy Checklist.

## Statistics

For all statistical analyses, confirm that the following items are present in the figure legend, table legend, main text, or Methods section.

| n/a | Confirmed | |
|---|---|---|
| ☐ | ☒ | The exact sample size (*n*) for each experimental group/condition, given as a discrete number and unit of measurement |
| ☐ | ☒ | A statement on whether measurements were taken from distinct samples or whether the same sample was measured repeatedly |
| ☒ | ☐ | The statistical test(s) used AND whether they are one- or two-sided<br>*Only common tests should be described solely by name; describe more complex techniques in the Methods section.* |
| ☒ | ☐ | A description of all covariates tested |
| ☒ | ☐ | A description of any assumptions or corrections, such as tests of normality and adjustment for multiple comparisons |
| ☒ | ☐ | A full description of the statistical parameters including central tendency (e.g. means) or other basic estimates (e.g. regression coefficient) AND variation (e.g. standard deviation) or associated estimates of uncertainty (e.g. confidence intervals) |
| ☒ | ☐ | For null hypothesis testing, the test statistic (e.g. *F*, *t*, *r*) with confidence intervals, effect sizes, degrees of freedom and *P* value noted<br>*Give P values as exact values whenever suitable.* |
| ☒ | ☐ | For Bayesian analysis, information on the choice of priors and Markov chain Monte Carlo settings |
| ☒ | ☐ | For hierarchical and complex designs, identification of the appropriate level for tests and full reporting of outcomes |
| ☒ | ☐ | Estimates of effect sizes (e.g. Cohen's *d*, Pearson's *r*), indicating how they were calculated |

*Our web collection on statistics for biologists contains articles on many of the points above.*

## Software and code

Policy information about availability of computer code

| | |
|---|---|
| Data collection | The FIB-SEM acquisitions are done using Zeiss Auriga with ATLAS 5 software. |
| Data analysis | The image processing are done using ImageJ v.1.52t, plugin Stacks - shuffling/Align Slices.The 3D images were analysed using 3D visualization software Amira version.2019.4. The segmentation is transfered to FIJI / ImageJ 1.52p running in JDK 1.8 environment. Processing, analysis and results plotting was done in Python environment: Python 3.6.6, skimage 0.14.2, matplotlib 2.2.2, seaborn 0.9.0, numpy 1.16.4, scipy 1.3.1, pandas 0.25.1. We used IDE (Integrated Development Environment) of Jupyter: jupyter core 4.5.0, jupyter-notebook 6.0.1, qtconsole 4.5.1, ipython 7.8.0, ipykernel 5.1.2, jupyter client 5.3.1, jupyter lab 1.1.4, nbconvert 5.5.0, ipywidgets 7.5.1, nbformat 4.4.0, traitlets 4.3. Graphical images shown in the manuscript were generated using ImageJ v.1.52t, Adobe Photoshop version 20.0.7 and Adobe Illustrator v.26.0.1, videos were generated using Amira version.2019.4 and Adobe Premiere Pro v.13.1.5. |

For manuscripts utilizing custom algorithms or software that are central to the research but not yet described in published literature, software must be made available to editors and reviewers. We strongly encourage code deposition in a community repository (e.g. GitHub). See the Nature Portfolio guidelines for submitting code & software for further information.

## Data

Policy information about availability of data

All manuscripts must include a data availability statement. This statement should provide the following information, where applicable:

- Accession codes, unique identifiers, or web links for publicly available datasets
- A description of any restrictions on data availability
- For clinical datasets or third party data, please ensure that the statement adheres to our policy

The 3D images and the image reconstructions generated and analysed during the current study are not publicly available due to size constrains but are available from the corresponding author on reasonable request. Exemplary electron micrographs and 3D reconstructions are represented in Figure 3. Source data for the statistical analyses are provided with this paper.

## Human research participants

Policy information about studies involving human research participants and Sex and Gender in Research.

| | |
|---|---|
| Reporting on sex and gender | not applicable |
| Population characteristics | not applicable |
| Recruitment | not applicable |
| Ethics oversight | not applicable |

Note that full information on the approval of the study protocol must also be provided in the manuscript.

# Field-specific reporting

Please select the one below that is the best fit for your research. If you are not sure, read the appropriate sections before making your selection.

☐ Life sciences ☐ Behavioural & social sciences ☒ Ecological, evolutionary & environmental sciences

For a reference copy of the document with all sections, see nature.com/documents/nr-reporting-summary-flat.pdf

# Ecological, evolutionary & environmental sciences study design

All studies must disclose on these points even when the disclosure is negative.

**Study description**

We performed 3D image analysis of the oocytes showing the intimate interaction between the intramitochondrial endosymbiont and tick oocytes at different vitellogenisation stages. We quantitatively describe the subcellular populations of endosymbionts and mitochondria, and accurately characterize their intersections from 4 wild-type and 2 aposymbiotic tick samples. The 3D reconstructions revealed new symbiosis-dependent morphologies of mitochondria in their full architecture.

**Research sample**

Wild-type and aposymbiotic European hard ticks, Ixodes ricinus, are used to obtain 3D images of the oocytes. The ovaries harbour the endosymbiont Midichloria mitochondrii that is maternally transmitted. Among the egg, larva, nymph and adult stages of ticks, the adults prior to oviposition had the highest numbers of endosymbiont. Therefore, we used the semi-engorged females to harvest ovaries that carry eggs in various vitellogenic stages. The images of the oocytes in different maturation stages were acquired to monitor the interaction with the endosymbiont-mitochondrion upon egg formation.
Approximately 3-5 weeks old wild-type females were collected from goats (Capra hircus) in a goat farm in Italy. The presence of the endosymbiont is confirmed by qPCR, TEM; prior to FIB-SEM imaging (n =4). The Neuchâtel line (i.e.: aposymbiotic ticks) has been reared on rabbits, and approximately 5-7 weeks old semi-engorged females are used. As the aposymbiotic ticks are very rare, no material could be used for qPCR. The absence of endosymbiont is proven by TEM; prior to FIB-SEM imaging (n = 2). Only vitellogenic and late vitellogenic stages of the posymbiotic ticks were observed to as engorgement and oviposition are drastically delayed compared to wild-type, and it was not possible to obtain a wide spectrum of maturating eggs.

**Sampling strategy**

No sampling procedure was used in this study, as all the samples were considered of interest due to challenges and limitations in the FIB-SEM image acquisition, and sparsity of the aposymbiotic ticks.

**Data collection**

Images were collected using Auriga Crossbeam Field Emission 481 Scanning Electron Microscope (Zeiss, Germany) of resin embedded samples. A 10 x 10 x 10 nm image resolution was aimed for each dataset with 2048 x 2048-pixel size in XY and milled on Z axis in 10 nm sections until the milling is interrupted. The SEM images were recorded with an aperture of 60 µm at 1.5 kV of the inlens EsB detector with the EsB grid set 488 to -300 to -500 V by Adeline Mallet and Zerrin Uzum. The images were aligned using Fiji, plugin Stacks - shuffling/Align Slices. Image reconstruction was done with original images using Amira v.2019.4 using manual segmentation tool by Zerrin Uzum. The segmentation data is transferred to Python 3.6 environment for image analysis by Dmitry Ershov ND Zerrin Uzum.

| | |
|---|---|
| Timing and spatial scale | Wild-type ticks were collected twice in September 2018 from Italy as ideal season for the adult engorgement, and the blocks were prepared in October-November 2018 in Paris, France. Aposymbiotic ticks were engorged once in laboratory in Nantes, France in February 2020 and the blocks were prepared in March 2020 in Paris, France. The image processing and reconstructions were performed between July 2018-July 2020 and the statistical analysis were finalized in June-August 2020, at Institut Pasteur, France. |
| Data exclusions | No data are excluded. |
| Reproducibility | In total four independent data acquisitions and image analyses for the wild-type ticks and two for the aposymbitoic ticks were performed. All the imaging were successful. The reproducibility of the FIB-SEM images were assessed using TEM by acquisition of random section from the same sample blocks. |
| Randomization | The wild-type female Ixodes ricinus ticks, and the aposymbiotic Neuchatel line were randomly selected into two experimental groups prior to the beginning of the experiments. Due to the small number of biological samples and 3D data acquisitions, no randomization was performed for the imaging and image analyses. |
| Blinding | Due to the small number of samples, no blinding was performed during sample preparation, date collection and image reconstruction. The authors were blinded during data analysis. |

Did the study involve field work? ☐ Yes ☒ No

# Reporting for specific materials, systems and methods

We require information from authors about some types of materials, experimental systems and methods used in many studies. Here, indicate whether each material, system or method listed is relevant to your study. If you are not sure if a list item applies to your research, read the appropriate section before selecting a response.

## Materials & experimental systems

| n/a | Involved in the study | |
|---|---|---|
| ☒ | ☐ | Antibodies |
| ☒ | ☐ | Eukaryotic cell lines |
| ☒ | ☐ | Palaeontology and archaeology |
| ☐ | ☒ | Animals and other organisms |
| ☒ | ☐ | Clinical data |
| ☒ | ☐ | Dual use research of concern |

## Methods

| n/a | Involved in the study | |
|---|---|---|
| ☒ | ☐ | ChIP-seq |
| ☒ | ☐ | Flow cytometry |
| ☒ | ☐ | MRI-based neuroimaging |

## Animals and other research organisms

Policy information about studies involving animals; ARRIVE guidelines recommended for reporting animal research, and Sex and Gender in Research

| | |
|---|---|
| Laboratory animals | Ixodes ricinus Neuchâtel line is maintained under laboratory condition upon which it is cleared from the symbiotic bacterial content over rearing. Approximately 5-7 weeks of adult semi engorged females were used for the experiments. Ticks were housed at 85% humidifying chambers at room temperature in regular light/dark cycle until dissection. Neuchâtel ticks were reared and engorged on 16 weeks-old, 3 kg heavy rabbit in the Oniris CRIP (Centre de Recherche et d'Investigation Préclinique) at BIOEPAR Laboratory, Nantes, France, agreement number E44271. The project APAFIS#19700-20190309v1 (Engorgement of Ixodes ricinus ticks on rabbits) . |
| Wild animals | Approximately 3-5 weeks of semi-engorged adult female Ixodes ricinus ticks were collected from goats (Capra hircus) in Northern Italy in September 2018. The collection was performed by an authorized veterinarian in the context of routine sanitary screenings. The ticks were shipped to Institut Pasteur in aerated plastic tubes at ambient temperature that is sealed biological sample shipment. Upon arrival, ticks were housed at 85% humidifying chambers at room temperature in regular light/dark cycle until dissection. |
| Reporting on sex | Females were used exclusively as the endosymbiont is located in ovaries. |
| Field-collected samples | No field-collected samples were used in this study. |
| Ethics oversight | Research on ticks does not require approval of an organization. The engorgement of ticks on rabbit was authorized was authorized by the CEEA-06 (Comité d'Ethique en Expérimentation Animale Pays de la Loire; Pays de la Loire Ethics Committee) (agreement number E44271). The collection of ticks from goats were performed in accordance with current Italian law and the European guidelines on the use of animals in science. |

Note that full information on the approval of the study protocol must also be provided in the manuscript.
