# Supplementary material for: Three-dimensional images reveal the impact of the endosymbiont Midichloria mitochondrii on the host mitochondria
Source: Nat Commun. 2023 Jul 12;14:4133. doi: 10.1038/s41467-023-39758-x (PMC10338478; doi:10.1038/s41467-023-39758-x)
Supplement: Supplementary file 2 — Peer Review File [file 41467_2023_39758_MOESM2_ESM.pdf]

nature portfolio

Peer Review File

Three-dimensional images reveal the impact of the endosymbiont *Midichloria mitochondrii* on the host mitochondria

Open Access This file is licensed under a Creative Commons Attribution 4.0 International License, which permits use, sharing, adaptation, distribution and reproduction in any medium or format, as long as you give appropriate credit to the original author(s) and the source, provide a link to the Creative Commons license, and indicate if changes were made. In the cases where the authors are anonymous, such as is the case for the reports of anonymous peer reviewers, author attribution should be to 'Anonymous Referee' followed by a clear attribution to the source work. The images or other third party material in this file are included in the article's Creative Commons license, unless indicated otherwise in a credit line to the material. If material is not included in the article's Creative Commons license and your intended use is not permitted by statutory regulation or exceeds the permitted use, you will need to obtain permission directly from the copyright holder. To view a copy of this license, visit http://creativecommons.org/licenses/by/4.0/.

REVIEWER COMMENTS

Reviewer #1 (Remarks to the Author):

Title: Three-dimensional images display the impact on the endosymbiont Midichloria mitochondrii on the host mitochondria

Authors: Zerrin Uzum, Dmitry Ershov, Adeline Mallet, Olivier Gorgette, Olivier Plantard, Davide Sassera, Fabrizia Stavru

This paper was reviewed by Vera Kozjak-Pavlovic and Christian Stigloher, University of Wuerzburg, Germany

Summary of the paper: This interesting study demonstrates the power of Focused Ion Beam-Scanning Electron Microscopy (FIB-SEM) for obtaining high-resolution 3D images, which enabled the authors to gain new insights into the relationship between mitochondria and endosymbiotic bacteria Midichloria mitochondrii known to come in contact and reside within the mitochondrial intermembrane space in the oocytes of the tick Ixodes ricinus. The authors rightfully identify the problems coupled with using Transmission Electron Microscopy (TEM) of thin sections – the correct categorization of bacteria-mitochondria interaction strongly depends on the area that has been sectioned, leading to a false-negative results. They therefore apply FIB-SEM to obtain 3D images of infected oocytes in several maturation stages, as well as compare the oocytes between wild type and aposymbiotic ticks, the latter being devoid of M. mitochondrii. Their analyses show that mitochondrial morphology and interaction with M. mitochondrii change throughout the oocyte maturation, with more bacteria being present in mitochondria at the later stages. Also, mitochondrial fragmentation was induced by the presence of M. mitochondrii. The authors describe novel phenotypes of mitochondria, which give rise to possible explanations of the bacterial life cycle.
In general, the experimental data is solid, and microscopy, as well as the quantification procedures are well performed. However, at certain points, the manuscript becomes too descriptive. Therefore, we would suggest some additional experiments that would strengthen the conclusions of the authors.
1.) Our primary concern is the description of the so called "reservoir mitochondrion". The authors describe only one such example, so all the other conclusions that they draw from this one observation are highly anecdotal. It would be good if more samples could be analyzed to try to identify more of such mitochondria. Understandably, FIB-SEM is not a method for such studies, but in this case, the classical TEM could be used to give at least some idea as to the frequency of such mitochondria harboring multiple bacteria. Likewise, the authors should tone down their conclusions regarding the morphology of the reservoir mitochondria since they were only able to observe one such mitochondrion.
2.) Along these lines, do the authors observe dividing bacteria within the mitochondria or in the cytosol? And what is the percentage of such dividing bacteria? Is it connected to the maturation stage, or do bacteria for example only divide within the mitochondria and never in the cytosol? This data should be provided if possible.
3.) Out of curiosity, what exactly is the reason that these oocytes cannot be studied using other microscopy techniques, such as fluorescence microscopy, or even expansion microscopy? Immunolabelling of such samples would enable better identification of bacteria and different mitochondrial membranes. Can the authors comment on this?
Regarding the structure of the manuscript, we would have some suggestions on how to improve the readability of the text.
4.) In the Introduction, it would be good if the authors could include some information on the FIB-SEM technique to prepare the readers for the rest of the story.
5.) Since the aposymbiotic tick play an important role for further experiments, it would be good to describe them at some point – how they are obtained, are they viable, what are the problems in their development that could be brought into the connection with the lack of M. mitochondrii.
6.) In the last sentence of the discussion the authors propose their images could form bases for further molecular studies that will deepen the understanding… Whereas the obtained 3D images are undoubtably valuable, it is primarily a technique as such that will offer tools for further studies of this and other similar bacteria-organelle interaction. Also, developing and introducing the

possibility to label the structures (bacteria, organelles, membranes) will for sure be necessary for further understanding of the processes. Perhaps the authors could point this out at the end of the discussion and maybe offer information if any such attempts have already been made.
7.) There are some sentences that should be rephrased or corrected:
- Lines 141 and 458: Carl Zeiss headquarters are given as Munich and Jena. If it's the same machine, the authors should decide where this particular part of Carl Zeiss is located.
- Line 143 "building up to a 3D-image" should be "building up to a 3D-image stack".
- Line 145 "(expected maximum resolution is 0.4 nm compared to 0.1 nm for TEM)" should add that this is the case for embedded biological samples.
- Line 209 "steadily reached to five" should be "steadily reached five".
- Line 293 "that resembles to a bacterial" should be "that resembles a bacterial".
- Line 329 "was associated organellar degradation" should be "was associated with organellar degradation".
- Line 330 "mitochondria remained." Intact perhaps? Or present?
- Lines 394/395 "the mitochondria might foster the endosymbiont in which M. mitochondrii can proliferate" sounds as if bacteria proliferate within an endosymbiont within the mitochondria. Maybe the sentence could be something like "mitochondria represent a niche in which M. mitochondrii can proliferate". Also, the rupture of reservoir mitochondrion does not cause cytoplasmic bacteria but is the source of cytoplasmic bacteria most likely.
- Line 448: in what were uranyl acetate and lead citrate dissolved. Water, methanol, or ethanol? Please specify.

Reviewer #2 (Remarks to the Author):

This is a superb study on the cellular biology of the intramitochondrial bacterium Midichloria mitochondrii and its tick host. It really was a pleasure to read. Since its description almost 20 years ago, the nature of the cellular interactions between this host-symbiont pair have remained ambiguous, partly due to the use of traditional TEM techniques.Using cutting edge microscopy techniques and a highly elegant experimental design, the authors of this study have made a number of key discoveries concerning this interaction, including the number of mitochondria infected in host cells, the number of symbionts present in mitochondria or in the cytoplasm, the different phenotypes the interaction may result in, and how bacterial numbers change during the development of oocytes. Their study is made particularly powerful through the use of aposymbiotic ticks, allowing inferences as to the major effects that M. midichloria has on mitochondrial structure in tick cells (i.e. severely breaking up networks of this organelle).

The authors hypothesise that the bacteria ensures its presence via endosymbiont regulated mitochondrial fragmentation. I think it would be worth being more explicit about the potential benefits to the host. It would be worth describing the fact that M. mitochondrii is found in essentially all female I. ricinus across its range, which suggests a mutualistic interaction. Do the authors think that the interaction is mutualistic, or is it possible that it is parasitic?

Some brief discussion of the results in relation to what is known from the genome of M. mitochondrii would also be worthwhile including if there is space.

The English expression of the manuscript is very good, and is easy to read, although it would benefit from proofreading and correction by a native English speaker.

In summary, I think this is a groundbreaking study that is worthy of publication in a top tier journal such as Nature Communications, following some minor revisions to the text.

Minor comment

Fig. 1
Don't "cross section III" images have some mitochondrial cristae at their edges, since they include mitochondria?

Reviewer #3 (Remarks to the Author):

Uzum et al., have investigated the oocytes of the tick I. ricinus with a focus on their mitochondria and the intracellular bacteria M. mitochondrii. They used FIB-SEM to obtain electron tomograms and performed quantitative analyses to conclude that (1) a large fraction of intracellular bacteria is physically associated, or found within, oocyte mitochondria, (2), the intracellular bacteria are found in the intermembrane space of oocyte mitochondria, (3) mitochondrial morphology depends on the presence of the intracellular bacteria, and (4) the number of intramitochondrial bacteria increases with oocyte development suggesting that M. mitochondrii reproduces inside mitochondria.

This is a technically sound study. The overarching goal is rather limited in scope: to investigate the spatial relationship between intracellular bacteria and mitochondria in the oocytes of the tick I. ricinus, and compare this across oocyte developmental stages and with aposymbiotic oocytes. A major motivation of this study was thus to confirm the idea that M. mitochondrii is fully found inside mitochondria, as previous two-dimensional electron micrographs were inconclusive. Additional important insights into the intracellular infection and oocyte development may have been obtained by combining the advanced microscopy employed here with (e.g., single-cell) transcriptomics. This may have provided more mechanistic insights.

In general, the readability of the manuscript has to be improved. For example, there is a lack of prepositions throughout the text, several misspellings (see below), and confusing sentences, all of which make the reading a little uncomfortable. Though the problems associated with the interpretation of 2D micrographs are well known, the authors perform simulations and quantitative analyses to justify their approach, which is welcomed.

General comments/questions:

Little is said about the intracellular bacterium M. mitochondrii in the introduction. It would be appreciated if it is introduced in the context of its phylogenetic affinities and closest relatives.

Mitochondria are well known to undergo fusion and produce mitochondrial networks such as those observed in aposymbiotic ticks in this study. Is it possible that mitochondrial fusion is partially a mechanism by which bacteria get inside mitochondria? One can imagine that an intracellular bacterium attaches to a mitochondrion, and that the latter envelopes the bacterium and fuses its membranes around it to effectively internalize it. This may be driven by machinery possessed by the bacterium, but the final fusion would involve mitofusins. Such a more 'passive' mechanism to enter mitochondria might also be consistent with the observations that the bacteria often lie parallel to the mitochondria as this might increase the surface area for membrane interactions. Are contact sites between the bacteria and mitochondria visible?

Are there any fitness consequences associated with harboring these intracellular symbionts in the oocytes? The mitochondrial phenotypes certainly go against what is often thought of as healthy and bioenergetically active mitochondria. These are often reticulated and have a high density of tight cristae. Are these intracellular/intramitochondrial bacteria found in 100% of individuals in tick populations? These aspects deserved discussion and might replace some of the content in the Discussion section that is largely redundant with the Results section.

The contrasting mitochondrial phenotypes observed in tick oocytes with and without intramitochondrial symbionts are similar to the 'condensed' and 'orthodox' morphologies associated with different energetic states of mitochondria (https://pubmed.ncbi.nlm.nih.gov/5968972/). When mitochondria become more active, they switch from an 'orthodox' state to a 'condensed' state with expanded crista lumen and contracted matrix (see also https://doi.org/10.1016/j.cub.2020.02.053). This is consistent with the notion that M. mitochondrii is an energy parasite and likely changes the ATP/ADP ratio of mitochondria forcing them to switch to the 'condensed' state. This might also explain the observation that globular mitochondria with swollen cristae are seen even when bacteria are not present within them. This topic deserves further discussion in the manuscript.

Do the findings of the authors support the idea that the intracellular bacteria have a somewhat pathogenic/parasitic relationship with their hosts? Little is concluded about the nature of the symbiotic relationship between I. ricinus and M. mitochondrii. The fact that M. mitochondrii in an energy parasite by virtue of bearing an ATP/ADP translocase might suggest this. Also, the disrupted mitochondria, presumably caused by the proliferation of M. mitochondrii and its exit, also supports this notion. Note that I'm not suggesting that M. mitochondrii is a 'full' or 'one-sided' parasite. Certainly, the fact that it is vertically inherited means that it has to somewhat align its interests with those of its tick host. But the evidence does not appear to suggest that M. mitochondrii is a benign mutualist. M. mitochondrii would certainly lie more in the parasitic region of the symbiotic spectrum.

Other comments:

Fig. 1. Label inner and outer membranes to support claim in L107-108.
L194: '…and *then* we *then*…'
L404: 'recruitment' instead of 'endorsement'
L101: Replace 'maturate' for 'mature'.
L373: Replace 'compressively' for 'comprehensively'.
L396: "*Previously*, the reservoir mitochondria were detected in *previous* TEM images"

Nature Communications

Three-dimensional images display the impact of the endosymbiont *Midichloria mitochondrii* on the host mitochondria
(Resubmission of revised MS, original Manuscript #: NCOMMS-22-09677)

Dear Reviewers,

Many thanks for taking your time and energy to evaluate our manuscript. Your enthusiastic feedback is much appreciated. We are also grateful for your insightful comments, which greatly helped improving this paper.

Please find our response to your valuable comments added below this letter. We hope that all points have been addressed to your satisfaction and that the manuscript is now ready for publication. On behalf of all coauthors, many thanks again for your support!

Sincerely,

Zerrin Uzum

Point-by-point response to reviewers' comments:

(*Response to the reviewer comments in italics*)

REVIEWER COMMENTS</B>

Reviewer #1 (Remarks to the Author):

Title: Three-dimensional images display the impact on the endosymbiont Midichloria mitochondrii on the host mitochondria

Authors: Zerrin Uzum, Dmitry Ershov, Adeline Mallet, Olivier Gorgette, Olivier Plantard, Davide Sassera, Fabrizia Stavru

This paper was reviewed by Vera Kozjak-Pavlovic and Christian Stigloher, University of Wuerzburg, Germany

Summary of the paper: This interesting study demonstrates the power of Focused Ion Beam-Scanning Electron Microscopy (FIB-SEM) for obtaining high-resolution 3D images, which enabled the authors to gain new insights into the relationship between mitochondria and endosymbiotic bacteria Midichloria mitochondrii known to come in contact and reside within the mitochondrial intermembrane space in the oocytes of the tick Ixodes ricinus. The authors rightfully identify the problems coupled with using Transmission Electron Microscopy (TEM) of thin sections – the correct categorization of bacteria-mitochondria interaction strongly depends on the area that has been sectioned, leading to a false-negative results. They therefore apply FIB-SEM to obtain 3D images of infected oocytes in several maturation stages, as well as compare the oocytes between wild type and aposymbiotic ticks, the latter being devoid of M. mitochondrii. Their analyses show that mitochondrial morphology and interaction with M. mitochondrii change throughout the oocyte maturation, with more bacteria being present in mitochondria at the later stages. Also, mitochondrial fragmentation was induced by the presence of M. mitochondrii. The authors describe novel phenotypes of mitochondria, which give rise to possible explanations of the bacterial life cycle.
In general, the experimental data is solid, and microscopy, as well as the quantification procedures are well performed. However, at certain points, the manuscript becomes too descriptive. Therefore, we would suggest some additional experiments that would strengthen the conclusions

of the authors.
1.) Our primary concern is the description of the so called "reservoir mitochondrion". The authors describe only one such example, so all the other conclusions that they draw from this one observation are highly anecdotal. It would be good if more samples could be analyzed to try to identify more of such mitochondria. Understandably, FIB-SEM is not a method for such studies, but in this case, the classical TEM could be used to give at least some idea as to the frequency of such mitochondria harboring multiple bacteria. Likewise, the authors should tone down their conclusions regarding the morphology of the reservoir mitochondria since they were only able to observe one such mitochondrion.

Response: *Thank you for your comment. Although, we could represent one reservoir mitochondrion in its full volume in vitellogenic oocyte, we could observe another one in partially in late vitellogenic oocyte. Also, such mitochondria were previously observed in TEM images. We have included examples of the reservoir mitochondrion observed in the other publications and tuned down the conclusions both in the introduction (line 87-91) and discussion (line 417-424), according to your valuable comment.*

2.) Along these lines, do the authors observe dividing bacteria within the mitochondria or in the cytosol? And what is the percentage of such dividing bacteria? Is it connected to the maturation stage, or do bacteria for example only divide within the mitochondria and never in the cytosol? This data should be provided if possible.

Response: *We strongly agree with this comment and, in fact, a special attention had been given to dividing bacteria. Based on your question, we added two sentences starting at Line 184 explaining the above.*

3.) Out of curiosity, what exactly is the reason that these oocytes cannot be studied using other microscopy techniques, such as fluorescence microscopy, or even expansion microscopy? Immunolabelling of such samples would enable better identification of bacteria and different mitochondrial membranes. Can the authors comment on this?

Response: *In parallel to the FIB-SEM, we performed live-staining the oocytes with MitoTracker and immunofluorescence with different commercially available antibodies. In our live-stained eggs, we could stain the nuclei with Hoechst, but we could not detect signal from any of the MitoTracker dyes (except the nuclear stain with MT orange). This is in line with the phenomenon observed in other examples in the animal kingdom that the mitochondria of the eggs are inactive to avoid delivery of ROS-exposed, hence mutated, mtDNA to the offspring. Additionally, the chorion of tick eggs is very hard for penetration of the dyes, and the lipoprotein content in the eggs is so high such that the permeabilization and clearing processes severely compromise the cellular spatial resolution. Lastly, we detected cross reactivity of one mammalian antibodies (*i.e.: *Tomm20) against tick cell lines, yet we failed to get any signal using this antibody against eggs. In the manuscript, we added a sentence that briefly summarized those attempts starting at line 371.*

In addition to this, we have performed mitochondrial staining to two tick cells lines and successfully observed the mitochondrial network. However, we did not find these two sets of data fundamentally relevant as the cell lines do not represent the situation in oocytes. We are willing to include these data to the manuscript if the reviewers find it useful.

Regarding the structure of the manuscript, we would have some suggestions on how to improve the readability of the text.
4.) In the Introduction, it would be good if the authors could include some information on the FIB-SEM technique to prepare the readers for the rest of the story.

Response: *Thanks for the suggestion. Three explanatory sentences are added regarding the FIB-SEM in introduction (Lines 94-97).*

5.) Since the aposymbiotic tick play an important role for further experiments, it would be good to describe them at some point – how they are obtained, are they viable, what are the problems in their development that could be brought into the connection with the lack of M. mitochondrii.

Response: *We thank to the reviewers for their highlight. The Neuchatel line has been initiated in 1978 in order to obtain tick population free of tick-transmitted pathogens and reared over many generations. Upon identification of* M. mitochondrii*, collaborations are established and different*

generations (namely F1, F4, F5 and F10) were tested. The females lost M. mitochondrii *in later generations and F10 has lost the endosymbiont completely. We have designed a quantitative approach to compare the WT and aposymbiotic populations, however, the aposymbiotic ticks are rare and sometimes die without engorgement, hence we could not collect enough data to comment on the fitness. Based on your comment, we have added this information in Lines 108-113.*

6.) In the last sentence of the discussion the authors propose their images could form bases for further molecular studies that will deepen the understanding… Whereas the obtained 3D images are undoubtably valuable, it is primarily a technique as such that will offer tools for further studies of this and other similar bacteria-organelle interaction. Also, developing and introducing the possibility to label the structures (bacteria, organelles, membranes) will for sure be necessary for further understanding of the processes. Perhaps the authors could point this out at the end of the discussion and maybe offer information if any such attempts have already been made.

Response: *Thank you for your sound comments, we have extensively revised the ending accordingly.*

7.) There are some sentences that should be rephrased or corrected:
- Lines 141 and 458: Carl Zeiss headquarters are given as Munich and Jena. If it's the same machine, the authors should decide where this particular part of Carl Zeiss is located.
- Line 143 "building up to a 3D-image" should be "building up to a 3D-image stack".
- Line 145 "(expected maximum resolution is 0.4 nm compared to 0.1 nm for TEM)" should add that this is the case for embedded biological samples.
- Line 209 "steadily reached to five" should be "steadily reached five".
- Line 293 "that resembles to a bacterial" should be "that resembles a bacterial".
- Line 329 "was associated organellar degradation" should be "was associated with organellar degradation".
- Line 330 "mitochondria remained." Intact perhaps? Or present?
- Lines 394/395 "the mitochondria might foster the endosymbiont in which M. mitochondrii can proliferate" sounds as if bacteria proliferate within an endosymbiont within the mitochondria. Maybe the sentence could be something like "mitochondria represent a niche in which M. mitochondrii can proliferate". Also, the rupture of reservoir mitochondrion does not cause cytoplasmic bacteria but is the source of cytoplasmic bacteria most likely.
- Line 448: in what were uranyl acetate and lead citrate dissolved. Water, methanol, or ethanol? Please specify.

Response: *All the points above have been addressed and all your valuable suggestions are integrated into the text.*

Reviewer #2 (Remarks to the Author):

This is a superb study on the cellular biology of the intramitochondrial bacterium Midichloria mitochondrii and its tick host. It really was a pleasure to read. Since its description almost 20 years ago, the nature of the cellular interactions between this host-symbiont pair have remained ambiguous, partly due to the use of traditional TEM techniques. Using cutting edge microscopy techniques and a highly elegant experimental design, the authors of this study have made a number of key discoveries concerning this interaction, including the number of mitochondria infected in host cells, the number of symbionts present in mitochondria or in the cytoplasm, the different phenotypes the interaction may result in, and how bacterial numbers change during the development of oocytes. Their study is made particularly powerful through the use of aposymbiotic ticks, allowing inferences as to the major effects that M. midichloria has on mitochondrial structure in tick cells (i.e. severely breaking up networks of this organelle).

The authors hypothesise that the bacteria ensures its presence via endosymbiont regulated mitochondrial fragmentation. I think it would be worth being more explicit about the potential benefits to the host. It would be worth describing the fact that M. mitochondrii is found in essentially all female I. ricinus across its range, which suggests a mutualistic interaction. Do the authors think that the interaction is mutualistic, or is it possible that it is parasitic?

Response: *We highly agree with the importance of addressing this overarching question and we expanded the discussion on the possible nature of the interaction. Indeed, coexistence at the cellular level indeed provokes such intriguing questions. Multipartite relationships are often*

described within a spectrum where mitochondria and plastids correspond to mutualistic end and the deadly infectious agents represent the pathogenic end. The beneficial symbionts in evolution in transition can be located at the mutualist end, while keeping their so-called pathogenic features for the sake of survival. Previously B. bacteriovorus*-like parasitism was proposed, yet the mitochondrial dynamic model, lack of mitophagy, maintained mitochondrial cristae and the successful propagation of the wild-type ticks compared to aposymbiotic ticks made us believe that* Midichloria mitochondrii *is a mutualistic bacterium. Following the interest by the reviewers we expanded the discussion on this topic starting at Line 389).*

Some brief discussion of the results in relation to what is known from the genome of M. mitochondrii would also be worthwhile including if there is space.

Response: *Thank you for your constructive comment. Indeed, we have another study focusing on the molecular analyses of the symbiosis factors where we combine the computational predictions with comparative genomics as well as functional analyses. We excluded those speculations in this study as we did not provide functional data and solely focused on the imaging. Yet, as a response to your review, we cited the other manuscript that is currently in review process and available at bioRxiv in Line 57-58.*

The English expression of the manuscript is very good, and is easy to read, although it would benefit from proofreading and correction by a native English speaker.

Response: *Thanks, we consulted Prof John M. Murray whose corrections we feel have greatly improved the quality of the text.*

In summary, I think this is a groundbreaking study that is worthy of publication in a top tier journal such as Nature Communications, following some minor revisions to the text.

Minor comment

Fig. 1
Don't "cross section III" images have some mitochondrial cristae at their edges, since they include mitochondria?

Response: *We respectfully believe the reviewer meant Fig. 2. If we understand correctly what the reviewer means, the cristae would not be seen, because the bacterium is located in the mitochondrial intermembrane space surrounded by the mitochondrial outer membrane. Depending on the location of cross section, the cristae might not be visible at all. For instance, Video 2 represents such scenario in which the earlier sections do not indicate any mitochondrial association, but the mitochondrion appears around 4th second. Similar situation can be seen on Fig6E, and the animated 3D view of the particular example.*

<u>Reviewer #3 (Remarks to the Author):</u>

Uzum et al., have investigated the oocytes of the tick I. ricinus with a focus on their mitochondria and the intracellular bacteria M. mitochondrii. They used FIB-SEM to obtain electron tomograms and performed quantitative analyses to conclude that (1) a large fraction of intracellular bacteria is physically associated, or found within, oocyte mitochondria, (2), the intracellular bacteria are found in the intermembrane space of oocyte mitochondria, (3) mitochondrial morphology depends on the presence of the intracellular bacteria, and (4) the number of intramitochondrial bacteria increases with oocyte development suggesting that M. mitochondrii reproduces inside mitochondria.

This is a technically sound study. The overarching goal is rather limited in scope: to investigate the spatial relationship between intracellular bacteria and mitochondria in the oocytes of the tick I. ricinus, and compare this across oocyte developmental stages and with aposymbiotic oocytes. A major motivation of this study was thus to confirm the idea that M. mitochondrii is fully found inside mitochondria, as previous two-dimensional electron micrographs were inconclusive. Additional important insights into the intracellular infection and oocyte development may have been obtained by combining the advanced microscopy employed here with (e.g., single-cell) transcriptomics. This may have provided more mechanistic insights.

Response:
Thank you for your detailed comment. Although we agree with referee 3 that transcriptomics would provide valuable and relevant data for a better understanding of the impact of Midichloria *on its host, such an experimental approach was out of the scope of our study. In particular, we agree that the expression level of genes involved in mitochondrial morphology and network are poorly known and are involved in a highly dynamic network of genes especially in an arthropod host that would be especially difficult to monitor at each stage of the mitochondrial "lifecycle".*

In general, the readability of the manuscript has to be improved. For example, there is a lack of prepositions throughout the text, several misspellings (see below), and confusing sentences, all of which make the reading a little uncomfortable. Though the problems associated with the interpretation of 2D micrographs are well known, the authors perform simulations and quantitative analyses to justify their approach, which is welcomed.

Response: *Thanks, we consulted Prof John M. Murray whose corrections we feel have greatly improved the quality of the text.*

General comments/questions:

Little is said about the intracellular bacterium M. mitochondrii in the introduction. It would be appreciated if it is introduced in the context of its phylogenetic affinities and closest relatives.

Response: *Thanks for the suggestion. A paragraph on this topic has been added in the introduction started at line 55.*

Mitochondria are well known to undergo fusion and produce mitochondrial networks such as those observed in aposymbiotic ticks in this study. Is it possible that mitochondrial fusion is partially a mechanism by which bacteria get inside mitochondria? One can imagine that an intracellular bacterium attaches to a mitochondrion, and that the latter envelopes the bacterium and fuses its membranes around it to effectively internalize it. This may be driven by machinery possessed by the bacterium, but the final fusion would involve mitofusins. Such a more 'passive' mechanism to enter mitochondria might also be consistent with the observations that the bacteria often lie parallel to the mitochondria as this might increase the surface area for membrane interactions. Are contact sites between the bacteria and mitochondria visible?

Response: *Thank you for kindly highlighting the possible mitochondrial colonization way. Our observations were limited to the adult females where we have always observed the intramitochondrial population, indicating that the mitochondrial colonization had already taken place. This could indicate that (i) mitochondrial colonization takes place before adult stages, or (ii) it happened once in evolutionary process in which* M. mitochondrii *remains in mitochondria throughout its life and occasionally leave mitochondria due to unknown reasons. Our snapshots support the latter, yet we do not have enough evidence to fully reject the mitochondrial colonization happening regularly. As we have discussed started in line 410, the 3D images imply a release of bacteria from the mitochondria due to the outwardly broken mitochondrial outer membrane of Pacman phenotype. On the other hand, we have observed a direct correlation between presence of endosymbiont and the mitochondrial fragmentation upon comparison WT and aposymbiotic tick where the mitochondrial dynamics could be manipulated by the bacteria. In this most plausible scenario,* M. mitochondrii *controls the mitochondrial fission upon formation of spherical oocyte mitochondria. Additionally, the resolution does not allow the visualization of the contact sites, and it would not be proven effectively with a label-free approach. In a different study, we had a genome-based approach to predict host and the bacterial genes that might be involved in the endosymbiosis. However, genetic manipulation is not possible either to the endosymbiont or the host and the labelling is tremendously hampered. As we cannot provide image-based evidences, we avoided genomics-based speculations to the manuscript.*

Are there any fitness consequences associated with harboring these intracellular symbionts in the oocytes? The mitochondrial phenotypes certainly go against what is often thought of as healthy and bioenergetically active mitochondria. These are often reticulated and have a high density of tight cristae. Are these intracellular/intramitochondrial bacteria found in 100% of individuals in tick populations? These aspects deserved discussion and might replace some of the content in the Discussion section that is largely redundant with the Results section.

Response:

Thank you very much for your valuable comment. It is highly desirable to be able to answer this question with evidence. In an independent study, we are gathering data on three different tick lines (WT with a symbiont, aposymbiotic and WT without an endosymbiont) and trying to characterize fitness cost (or benefit) in the presence and absence of Midichloria *on ticks. Although it fails outside the scope of this manuscript, we appreciate the interest of the reviewer 3, and share our primitive data. Engorging ticks experimentally is a highly variable process (resulting from the interaction between the vertebrate host used for the blood meal, including its immune reaction and its physiological state), and the as well as varying feeding times such that it is hard to observe statistical differences. Although we have detected a low performance of the aposymbiotic ticks upon rearing, we could not have enough samples to perform quantitative analyses as we can have very few of aposymbiotic ticks. However, in this manuscript, a clear evidence is presented on the mitochondrial morphologies having a strong correlation with mitochondrial tropism. We have added this information in the manuscript and significantly reduced the redundancies in the discussion according to your comment.*

The contrasting mitochondrial phenotypes observed in tick oocytes with and without intramitochondrial symbionts are similar to the 'condensed' and 'orthodox' morphologies associated with different energetic states of mitochondria (https://pubmed.ncbi.nlm.nih.gov/5968972/). When mitochondria become more active, they switch from an 'orthodox' state to a 'condensed' state with expanded crista lumen and contracted matrix (see also https://doi.org/10.1016/j.cub.2020.02.053). This is consistent with the notion that M. mitochondrii is an energy parasite and likely changes the ATP/ADP ratio of mitochondria forcing them to switch to the 'condensed' state. This might also explain the observation that globular mitochondria with swollen cristae are seen even when bacteria are not present within them. This topic deserves further discussion in the manuscript.

Response:
Thank you very much for the constructive comments and literature suggestions. As you have nicely stated, the mitochondrial morphology as well as the fragmentation is correlated with the ATP production and cristae varies among eukaryotes in examples you have kindly shared. Although the mitochondria of mammals and arthropods have a lot in common, there are many differences especially when it comes to oocytes. Oocytes are responsible for delivery of healthy mitochondrial DNA to the offspring, hence very often they pack an inactive "progeny" of mitochondria often in sub-oocyte compartment called Balbiani body. Some species do not show any Balbiani body in oocytes and they keep the mitochondria completely inactive and obtain ATP through nurturing cells. To our knowledge, no such compartmentalization has been documented for arthropods. Also, the mitochondria of many species appear globular in these stages for easy distribution. Although this fact is not clearly documented for tick oocytes or arthropods in general, TEM images of other tick species shown to have globular mitochondria in oocytes (line 380). The oocyte mitochondrial phenotypes indeed look unhealthy and bioenergetically inactive, as they have different function than in cell lines handled in vitro*. We have included additional information and citations to explain the phenomenon further.*

Do the findings of the authors support the idea that the intracellular bacteria have a somewhat pathogenic/parasitic relationship with their hosts? Little is concluded about the nature of the symbiotic relationship between I. ricinus and M. mitochondrii. The fact that M. mitochondrii in an energy parasite by virtue of bearing an ATP/ADP translocase might suggest this. Also, the disrupted mitochondria, presumably caused by the proliferation of M. mitochondrii and its exit, also supports this notion. Note that I'm not suggesting that M. mitochondrii is a 'full' or 'one-sided' parasite. Certainly, the fact that it is vertically inherited means that it has to somewhat align its interests with those of its tick host. But the evidence does not appear to suggest that M. mitochondrii is a benign mutualist. M. mitochondrii would certainly lie more in the parasitic region of the symbiotic spectrum.

Response:
Thank you for highlighting the relationship with constructive suggestions. Indeed, it is very valuable yet challenging to ascribe an intracellular existence to be beneficial without molecular evidences. As you have nicely pointed, M. mitochondrii *cannot be described as full or one-sided parasite and the inheritance indicate the beneficial interaction. We have added further discussion starting in line 392 giving examples of the endosymbiont in transition and remaining pathogenic/parasitic features to manipulate the host to maintain the beneficial interaction.*

Other comments:

Fig. 1. Label inner and outer membranes to support claim in L107-108.
L194: '…and *then* we *then*…'
L404: 'recruitment' instead of 'endorsement'
L101: Replace 'maturate' for 'mature'.
L373: Replace 'compressively' for 'comprehensively'.
L396: "*Previously*, the reservoir mitochondria were detected in *previous* TEM images"

Response: *Your valuable suggestions are integrated into the text.*

REVIEWERS' COMMENTS

Reviewer #1 (Remarks to the Author):

The revised version of the manuscript has addressed all my points in a satisfactory manner. I support the publication of the manuscript in the present form.

Reviewer #2 (Remarks to the Author):

The authors have responded adequately to my comments. I believe the manuscript is now acceptable for publication.

Reviewer #3 (Remarks to the Author):

I consider the revised manuscript acceptable for publication. I have no further specific comments.

Nature Communications

Three-dimensional images reveal the impact of the endosymbiont *Midichloria mitochondrii* on the host mitochondria
(Resubmission of revised MS, original Manuscript #: NCOMMS-22-09677B)

Dear Reviewers,

Many thanks for taking your quick response to revise our manuscript. Your kind consent is much appreciated. We are again grateful for your insightful comments, which greatly helped improving this paper. On behalf of all coauthors, many thanks again for your support!

Sincerely,

Zerrin Uzum
